# A causal inference framework for identifying essential genes to enhance drug synergy prediction

**DOI:** 10.1093/bioinformatics/btag010

**Published:** 2026-05-28

**Authors:** Huaiwu Zhang, Xinliang Sun, Jianxin Wang, Min Li, Jing Tang

**Affiliations:** Research Program in Systems Oncology, Faculty of Medicine, University of Helsinki, Helsinki, 00290, Finland; School of Computer Science and Engineering, Central South University, Changsha, 410083, China; School of Computer Science and Engineering, Central South University, Changsha, 410083, China; School of Computer Science and Engineering, Central South University, Changsha, 410083, China; Research Program in Systems Oncology, Faculty of Medicine, University of Helsinki, Helsinki, 00290, Finland

## Abstract

**Motivation:**

Identifying synergistic drug combinations holds promise for more effective treatment strategies. Recent deep learning methods such as Transformers and Graph Neural Networks have shown improved predictive performance, but most of them integrate drug and cell line representations without explicitly modelling the causal effects of genes in mediating drug responses.

**Results:**

We introduce CADS (Causal Adjustment for Drug Synergy), a deep learning framework that explicitly models the gene–drug causal relationships to improve both prediction accuracy and biological interpretability. CADS integrates multi-omics data with a learnable gene-selection mechanism that performs causal backdoor adjustment, enabling both drug synergy prediction and causal gene discovery. Across multiple benchmark datasets, CADS consistently achieves superior performance compared with state-of-the-art drug synergy prediction models. In addition, downstream analyses on case studies demonstrate that the inferred gene causal scores can recover clinically validated cancer-related genes involved in drug combinations. These results demonstrate that explicitly modelling causal genetic effects can enhance the reliability and interpretability of drug synergy prediction.

**Availability and implementation:**

The source code of CADS can be found at https://github.com/HuaiwuZhang/causalDC.

## 1 Introduction

Combination therapies have become a promising strategy for the treatment of complex diseases such as cancer and infectious diseases (Al-Lazikani *et al.* 2012). By simultaneously perturbing multiple biological pathways, drug combinations can improve therapeutic efficacy and reduce the emergence of drug resistance compared to monotherapies (Gilad *et al.* 2021). Despite these advantages, identifying truly synergistic drug pairs remains challenging, as most clinically-approved combinations exhibit additive or independent effects rather than biologically meaningful interactions (Bulusu *et al.* 2016, Plana *et al.* 2022).

Experimental assay of drug combinations is commonly performed using high-throughput screening (HTS) platforms. Although effective on a small scale, HTS becomes increasingly infeasible as the combinatorial search space grows exponentially (Sadybekov and Katritch 2023). Furthermore, HTS screens often provide limited insight into the molecular mechanisms underlying drug interactions, thereby restricting their utility for rational drug design and clinical translation (Broach and Throner 1996). These limitations have motivated the development of computational approaches that can efficiently prioritize candidate drug combinations while offering mechanistic interpretations, thus establishing causal links with biological pathways and biomarkers.

With the increasing availability of large-scale pharmacogenomics resources such as DrugComb (Zagidullin *et al.* 2019), computational prediction of drug synergy has advanced rapidly. Early studies often relied on classical machine learning models, including random forests (Jeon *et al.* 2018), support vector machines (SVMs) (Noble 2006), and gradient-boosting methods (Chen and Guestrin 2016), which integrated features such as drug chemical descriptors and genomic profiles of cell lines (Wu *et al.* 2022). For instance, ERT (Jeon *et al.* 2018) integrated monotherapy responses and synthetic lethality data with random forests and SVMs to predict anticancer synergy, while DCPT (He *et al.* 2018) leveraged XGBoost to model selective synergy based on compound sensitivity differences. While these approaches demonstrated improved results, their performance often degraded when applied to unseen drug combinations or untested biological contexts, reflecting limited generalization capability (Partin *et al.* 2023).

More recently, deep learning methods have emerged as the dominant paradigm for drug synergy prediction, achieving improved predictive accuracy across multiple benchmarks (Ahmed *et al.* 2023). Representative methods such as DeepSynergy (Preuer *et al.* 2018) and MatchMaker (Kuru *et al.* 2022) introduced multi-branch neural architectures that jointly encode drug fingerprints and gene expression profiles. Subsequent work expanded this framework by incorporating drug-induced transcriptomic responses (EC-DFR (Lin *et al.* 2022)), graph neural networks for molecular structure modeling (DeepDDS (Wang *et al.* 2022)), hypergraph representations of drug–cell interactions (HypergraphSynergy (Liu *et al.* 2022)), and biological prior knowledge such as drug–target and protein–protein interaction networks (Jiang *et al.* 2020; Yang *et al.* 2021; Yu *et al.* 2022).

To further enhance the prediction performance, recent studies have focused on cross-modal interaction modeling, using attention mechanisms and Transformer-based architectures to capture complex dependencies between drugs, genes, and cellular contexts (Hu *et al.* 2022, Xu *et al.* 2023, Rafiei *et al.* 2023). In particular, SynergyX incorporates dynamic cross-modal interaction modeling together with biological network information (Guo *et al.* 2024), while PermuteDDS adopts a complementary strategy by constructing rich, multi-dimensional drug representations from diverse fingerprint types (Zhao *et al.* 2024). More recently, DVHSyn learns drug–drug–cell triplet context on a hypergraph and its expanded heterogeneous graph with selective fusion (Yu *et al.* 2026), while MDI-DDI integrates both the 2D and 3D structural information of drugs (Gong *et al.* 2025). In addition, MOSAIC splits SMILES into meaningful fragments and performs multi-granularity prediction (Zhang *et al.* 2025), and BAITSAO (Liu *et al.* 2025) uses LLM-derived drug and cell-line embeddings together with multi-task pre-training on large synergy datasets. Despite these methodological advances, two fundamental challenges remain unresolved. First, many models do not explicitly distinguish causal genetic factors from spurious correlations introduced by the high-dimensionality of cell line molecular features, leading to unstable predictions across datasets. Second, most existing approaches focus primarily on predictive performance, offering limited interpretability about which genes causally affect drug synergy.

In this study, we proposed CADS, a deep learning framework to explicitly model the causal effects of genes in drug synergy. CADS integrates multiple drug representations, including SMILES strings and chemical fingerprints, with multi-omics cell line profiles through a novel multi-modal fusion strategy. By constructing a structural causal model and applying backdoor adjustment via a learnable masking mechanism, CADS separates causal genes from confounding genetic factors during representation learning. This design enables the model to achieve higher predictive accuracy while simultaneously identifying genes that are causally relevant to drug interactions. A preliminary version of this study has appeared in the ISBRA 2025 conference (Zhang and Tang 2025). Compared to the conference version, we have made major extensions including:

- We incorporated CADS into multiple baselines and conducted a more comprehensive evaluation of predictive performance and robustness.
- We performed an interpretability analysis which revealed that embedding CADS strengthens cell line data representations to improve performance across various metrics.
- We highlighted the capability of CADS to uncover novel drug combinations and drug targets through substantial case studies.

**Figure 1:**
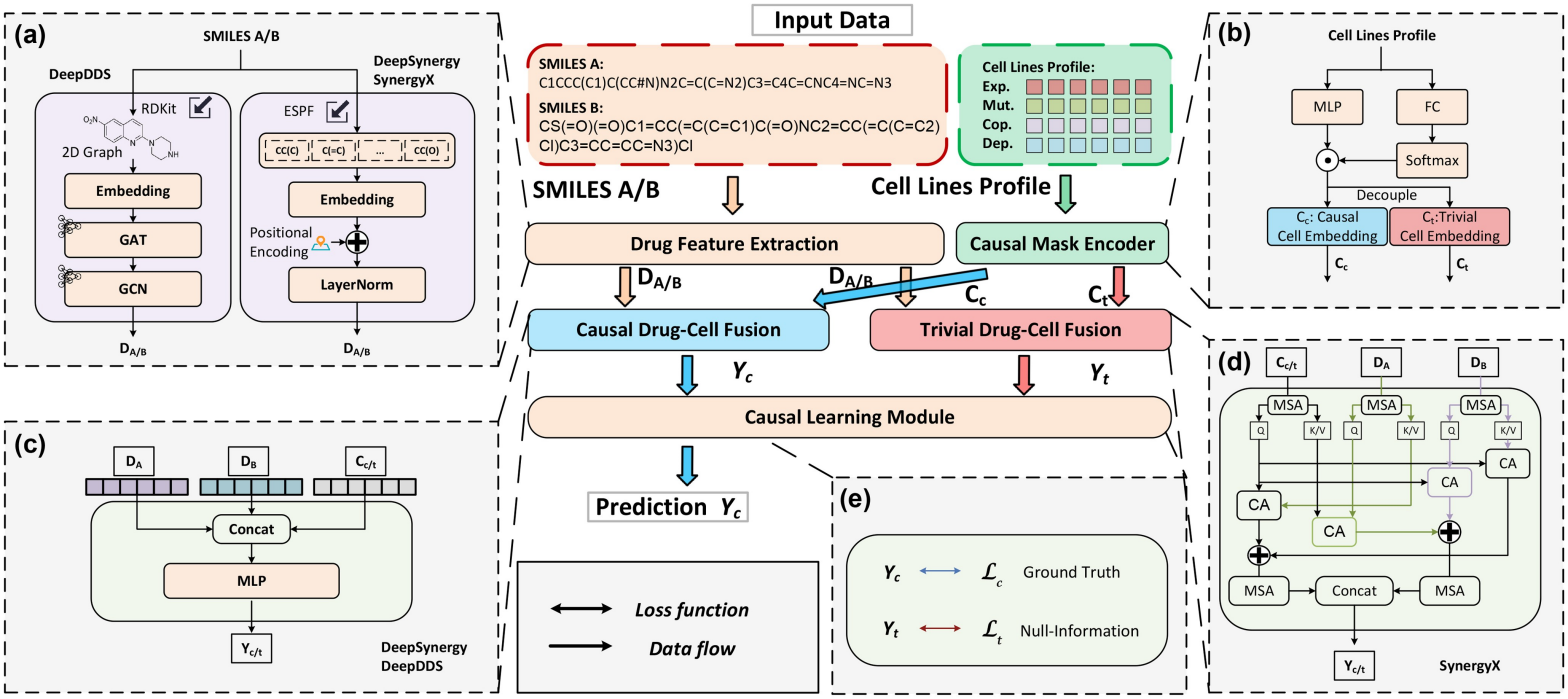
The pipeline of CADS. The CADS framework primarily consists of three main components: (a) *Drug Feature Extraction* is employed to extract drug features from sequences or 2D molecular graphs; (b) *Causal Mask Encoder* generates learnable causal mask pairs to decouple causal information from trivial information leveraging cell line profiles; (c–d) *Drug-Cell Fusion* employs Multi-Layer Perceptrons (MLP) or Cross-Attention (CA) and Multi-head Self-Attention (MSA) to fuse drug features with decoupled cell representations; (e) *Causal Learning Module* learns hard-to-observe causal information by training the causal and trivial components separately with ground truth and null-information labels, respectively.

The paper is organized as follows. In Section 2 we began with causal inference by constructing a *structural causal model* (SCM) (Figure 1, available as supplementary data at *Bioinformatics* online), and then designed a deep learning framework for the prediction of drug synergy. In Section 3 we integrated CADS into several carefully selected baseline models and demonstrated its predictive performance through comprehensive experiments. We then conducted ablation and few-shot analyses to validate the CADS’s performance and robustness, together with multiple case studies to identify and interpret causal genes associated with drug synergy. Section 4 provides a detailed discussion of the results and the limitations of the proposed approach.

## 2 Methodology

In this section, we presented the details of our CADS framework. We introduced CADS, a novel deep learning module designed to explicitly model causal relationships in drug-cell interactions. This component is systematically embedded into three state-of-the-art baseline frameworks including: DeepSynergy (Preuer *et al.* 2018), DeepDDS (Wang *et al.* 2022), and SynergyX (Guo *et al.* 2024).

### 2.1 Framework overview

Figure 1 shows the framework of CADS. Previous methods for drug synergy prediction typically employ SMILES sequence (Guo *et al.* 2024) or 2D graph (Wang *et al.* 2022) to represent global features of drugs, followed by feature concatenation with cell line characteristics or feature fusion using a cross-attention mechanism. It is noteworthy that in drug synergy prediction, when specific drug combinations are regarded as the primary entity, the intercellular contextual information *C* corresponding to each sample is incorporated into the encoding process as auxiliary information. To enhance conciseness of notation, we denoted this encoding process as F throughout this paper.

As illustrated in Appendix Section C, available as supplementary data at *Bioinformatics* online, the sole extraction of cell line information may introduce potential confounding factors. Therefore, when modeling drug combinations and cell line representations, we adopted a causal decoupling strategy: decomposing the encoder F into two parallel branches-a causal encoder $F_{c}$ and a trivial encoder $F_{t}$, which maintain identical architectures but possess non-shared parameters. This process can be formally represented as follows:


(1) $$\begin{array}{c} H_{c}=F_{c}(D_{A},D_{B};\;G_{c}),\;\;H_{t}=F_{t}(D_{A},D_{B};\;G_{t}), \end{array}$$

Where $G_{c}$ and $G_{t}$ represent causal cell information and trivial cell information. The embeddings $H_{c}$ and $H_{t}$ represent the final encoded representations of drug pairs after integrating causal information and trivial information, respectively.

Next, under the guidance of a specifically designed *Causal Mask Mechanism*, the output features of the two encoders are disentangled through separate processing pathways. $H_{c}$ and $H_{t}$ are fed into dual processing paths:

- *Causal branch*: Extracts invariant representations (causal features) from $H_{c}$.
- *Trivial branch*: Captures confounding effects (trivial features) from $H_{t}$.

**Figure 2:**
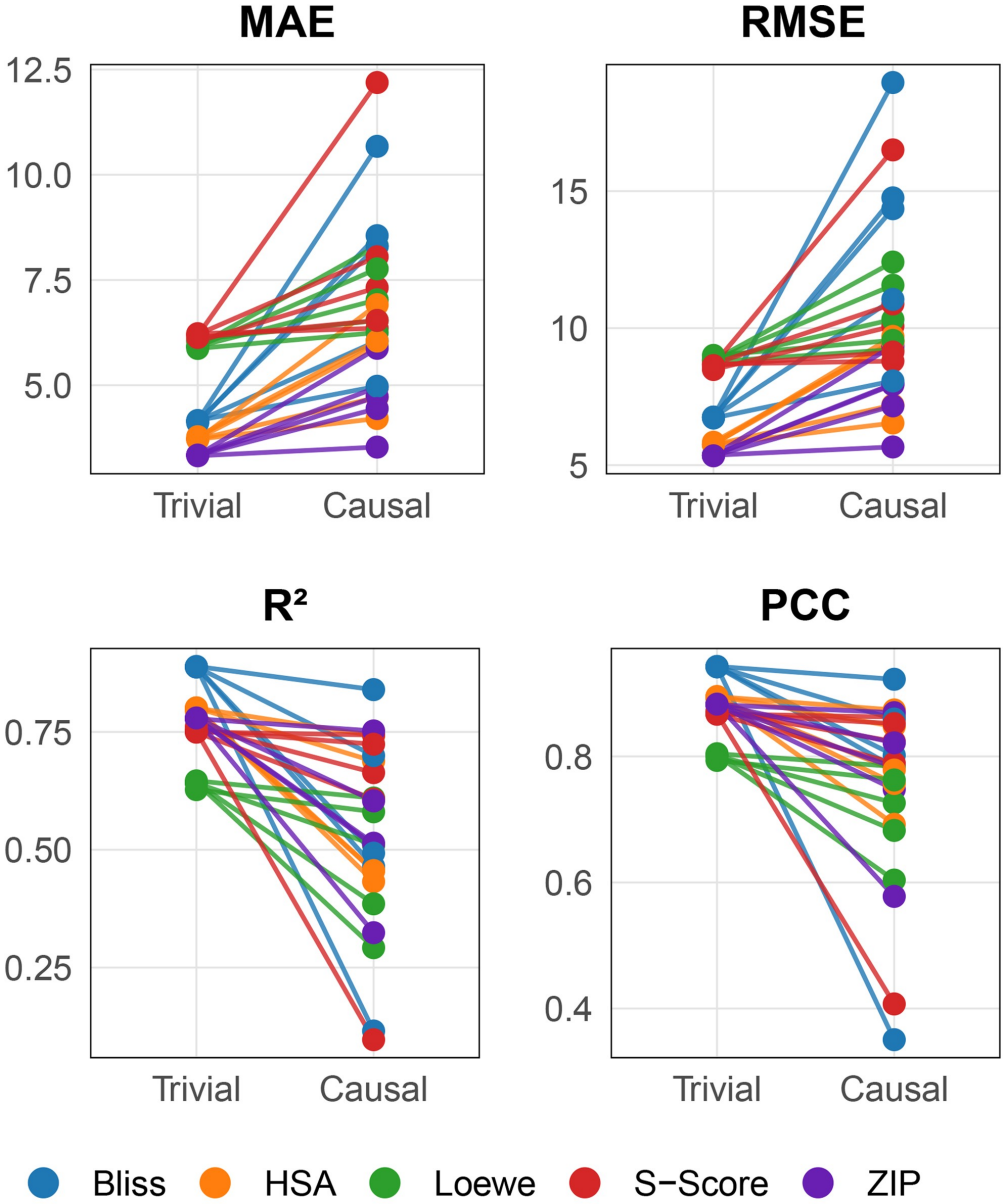
The *Paired Comparison Plot* illustrates the performance losses in four evaluation metrics (MAE, RMSE, $R^{2}$, PCC) for five synergy metrics (*Bliss*, *HSA*, *Loewe*, *S-Score*, *ZIP*) following the zero-masking of the top 1000 trivial genes and the top 1000 causal genes.

Finally, a *Causal Learning Module* subsequently performs representation-level interventions to coordinate with downstream tasks, enabling explicit separation between causal features and confounding factors to achieve robust representation learning. We further explained and illustrated the changes to the model architecture before and after CADSembedding in Appendix Section D and Fig. 2, available as supplementary data at *Bioinformatics* online to make them easier to visualize.

In the subsequent sections, we presented comprehensive details on the design of key modules and their integration methodology into the baseline framework.

### 2.2 Drug feature extraction

As shown in Fig. 1a, this module performs feature extraction on drug SMILES sequences. Due to the distinct drug representation handling mechanisms across the selected baseline models (Sequence or 2D-graph), we implemented two separate sets of feature extraction methodologies following prior research. As illustrated in Appendix Section E.2.1, available as supplementary data at *Bioinformatics* online, for SynergyX and DeepSynergy which process sequential structures, we segmented extended SMILES sequences into interpretable substructures using tokenization methodologies analogous to those in natural language processing (Huang *et al.* 2019), with each substructure treated as a discrete token (T):


(2) $$D_{k}=N(T⊕P),\;k\in K:=\{A,B\},$$

Where $D_{k}$ is the representation of Drug A or Drug B, N means layer normalization, P represents the positional encoding matrix satisfying $∥P_{i:}{∥}_{2}=1$.

For DeepDDS processing 2D molecular graph structures, SMILES representations were converted into graph topologies using the RDKit library, followed by cascaded GAT (A) (Veličković *et al.* 2017) and GCN (G) (Kipf and Welling, 2018) architectures to capture local and global graph features respectively:


(3) $$D_{k}=G{}^{\circ}A\left(V^{\left(k\right)},E^{\left(k\right)}\right),\;k\in K:=\{A,B\}$$

Where $V^{\left(k\right)}$ and $E^{\left(k\right)}$ represents denote the set of atoms and chemical bonds, respectively.

### 2.3 Causal mask encoder

Concurrently with drug feature extraction, feature encoding of cell lines was performed in parallel. Appendix Section C.1, available as supplementary data at *Bioinformatics* online highlights that filtering out trivial genes is essential for uncovering true causal impacts in the context of drug synergy. Nevertheless, explicitly distinguishing between causal and trivial genes remains a significant challenge in real-world scenarios. To overcome this limitation, we developed a computational framework designed to refine parametric masks via discriminative learning. Specifically, we employed a *causal awareness mask* ($M_{c}$) alongside a *trivial awareness mask* ($M_{t}$) to direct the distinct decoupling of features throughout parallel branches. For any given cell line profile $C\in R^{F\times N}$, a soft mask $M\in R^{1\times N}$ was allocated. Within this mask, individual elements denote the relative significance of their respective genes, generally scaled to a range of 0–1. Given a mask M, its complementary mask is defined as


(4) $$\bar{M}=1_{N}-M,$$

Where $1_{N}$ denotes an all-ones matrix. In formal terms, the collection of all causal genes constitutes the causal gene set, whereas the remaining involved genes are grouped into the trivial gene set. To learn these masks through the cell lines information while ensuring traceability to individual genes, we first performed feature extraction from cell lines using a fully-connected neural network. For each gene $g_{i}$, we computed its attention scores as follows:


(5) $$\begin{array}{cc} \alpha_{i}=\left[\begin{array}{c} \frac{e^{{\left(Wg_{i}\right)}_{1}}}{e^{{\left(Wg_{i}\right)}_{1}}+e^{{\left(Wg_{i}\right)}_{2}}}\\ \frac{e^{{\left(Wg_{i}\right)}_{2}}}{e^{{\left(Wg_{i}\right)}_{1}}+e^{{\left(Wg_{i}\right)}_{2}}} \end{array}\right]=\left[\begin{array}{c} \alpha_{c_{i}}\\ \alpha_{t_{i}} \end{array}\right] & \\ \text{for}\;i=1,\ldots ,N & \end{array}$$

Where $\alpha_{c_{i}}$ and $\alpha_{t_{i}}$ denote the attention weights at the gene level for a specific gene $g_{i}$,corresponding to the causal and trivial masks, respectively. We apply a softmax across the two-channel vector for each gene *i*, yielding gene-level attention scores $\alpha_{c_{i}}$ and $\alpha_{t_{i}}$ for the causal and trivial masks, respectively. By construction, this ensures $\alpha_{c_{i}}+\alpha_{t_{i}}=1$. These attention scores indicate the model’s attention to each gene within the corresponding cell-line feature matrix. We then construct a set of soft masks based on these scores:


(6) $$M_{c}={\left[\alpha_{c_{i}}\right]}_{i=1}^{N},\;M_{t}={\left[\alpha_{t_{i}}\right]}_{i=1}^{N},\;M_{c}+M_{t}=1_{N}$$

Therefore, our primary goal was to optimize the learnable representations $G_{c}=C⊙M_{c}$ and $G_{t}=C⊙M_{t}$ for the effective separation of causal and trivial genes, with ⊙ indicating the Hadamard product.

Up to now, we have captured causal features within cell line representations by allocating gene-level attention scores. Besides drug and cell representations ($D_{A/B}$; $G_{c/t}$) have been mapped into a shared feature space through the projection process.

### 2.4 Drug-cell fusion

In this section, we introduced a dual-branch parallel architecture (Causal/Trivial Drug-Cell Fusion) to implement cross-modal alignment between pharmacological data and cellular gene profiles, thereby connecting drug features with causal/trivial gene modalities respectively through inter-modal interactions.

As shown in Fig. 1d, for SynergyX, we further employ a set of cross-attention (CA) and multihead self-attention (MSA) mechanisms:


(7) $$\begin{array}{c} CA(X_{1},X_{2})=\sigma \left(\frac{(X_{1}W_{h}^{Q}){\left(X_{2}W_{h}^{K}\right)}^{⊤}}{\sqrt{d_{k}}}\right)(X_{2}W_{h}^{V}),\\ MSA(X)=\sigma \left(\frac{(XW_{h}^{Q}){\left(XW_{h}^{K}\right)}^{⊤}}{\sqrt{d_{k}}}\right)(XW_{h}^{V}), \end{array}$$

Where σ(·) denotes the softmax function. First, cross-modal communication is executed between the drug and cell line representations:


(8) $$\begin{array}{c} G_{i}^{\prime }=CA(G_{i},D_{k}),\\ D_{k}^{\prime }=CA(D_{k},G_{i});\\ G_{i}=G_{i}^{\prime }\\ \;i\in \{c,t\},\;k\in \{A,B\} \end{array}$$

Furthermore, recognizing that drug synergy is deeply governed by underlying pharmacological and pharmacokinetic mechanisms, we systematically model the interactive features between the two drugs, which is a crucial step:


(9) $$\begin{array}{c} D_{i}=CA(D_{i}^{\prime },D_{k}^{\prime });\;i,k\in K:=\{A,B\},\;i\neq k \end{array}$$

For i,k∈K=A,B with i≠k, we apply cross-attention in both directions: $D_{A}=CA(D_{A}^{\prime },D_{B}^{\prime })$ and $D_{B}=CA(D_{B}^{\prime },D_{A}^{\prime })$; thus $D_{A}^{\prime }$ attends to $D_{B}^{\prime }$ and vice versa, and both directions are used. Next, for all three baseline models, we adhered to the original methodology by concatenating $(||)D_{A/B}$with $G_{c/t}$, followed by MLP-based prediction $Y_{i}$ in our implementation:


(10) $$Y_{i}=\varphi {}^{\circ}\text{MLP}(G_{i}||D_{A}||D_{B}),\;i\in \{c,t\}$$

Where φ represents the Tanh(·) activation functions.

### 2.5 Causal learning module

Through the previously established dual-branch parallel architecture, we derived corresponding drug synergy score predicted based on Causal and Trivial genes, respectively ($Y_{i}$, $Y_{i}$). Considering that it is difficult to manually identify causal versus trivial genes discussed in Appendix Section C.2, available as supplementary data at *Bioinformatics* online, we leveraged the causal masking mechanism described in Section 2.3 to conduct joint training, thereby achieving gene disentanglement between Causal and Trivial genetic components.

As established by the Structural Causal Model (SCM), causal genes exert genuine influence on drug synergy. Consequently, the output of the causal branch undergoes regression training using ground-truth labels (${\hat{Y}}_{c}$). In contrast, trivial genes are designed to encapsulate mechanisms orthogonal to drug synergy. Therefore, the trivial branch is trained with all-zero values as null-information labels (${\hat{Y}}_{t}$), with the objective of rendering this branch incapable of discriminating between synergistic and antagonistic effects. Consequently, we defined the loss functions as follows:


(11) $$\begin{array}{c} L=H(Y_{c},{\hat{Y}}_{c})+H(Y_{t},{\hat{Y}}_{t}),\;{\hat{Y}}_{t}=0, \end{array}$$

With H(·,·) denoting the standard Mean Squared Error (MSE) loss function. By leveraging backpropagation, we imposed stability conditions on the causal genes and independence requirements on the trivial ones. This strategy effectively mitigates spurious correlations, thereby yielding more reliable drug synergy predictions.

## 3 Experiments & results

### 3.1 Overall performance

Table 1 presents the overall performance across five distinct scores (See also Appendix Section E.2.2, available as supplementary data at *Bioinformatics* online) under varying model configurations. Specifically, we reported the performance results for the original baseline model and the model employing the CADS framework (+CADS). The experimental results reveal a clear overarching trend: performance increased across all three baseline models following CADS integration, with a more pronounced improvement observed in the architecturally more complex SynergyX model. Notably, in the predictive performance assessment using multiple scores (*i.e.*, Loewe, HSA), the simpler DeepSynergy architecture outperformed the more complex SynergyX prior to CADS embedding. However, post-CADS integration, the performance of the complex SynergyX model surpassed that of the simpler model. A critical observation emerges when comparing how the two backbone models benefit from CADS. While DepSynergy+CADSshowed performance gains, these improvements were statistically significant in only a subset of the evaluation metrics (specifically Loewe and ZIP). In contrast, the integration of CADS into SynergyX resulted in statistically significant improvements (p<0.05) across a much broader array of indicators simultaneously. This phenomenon highlights a key interaction between model complexity and causal constraints. SynergyX, with its Transformer-based architecture, possesses a significantly larger parameter space and higher model capacity than the MLP-based DeepSynergy. While this high capacity allows SynergyX to capture intricate molecular patterns, it also renders the model more susceptible to learning spurious correlations, which can lead to inconsistent performance across different evaluation dimensions. This suggests that complex models may be prone to overfitting cell line contexts, potentially introducing additional confounding factors. In contrast, embedding CADS effectively disentangles these confounding factors, thereby enabling the complex model to realize its superior performance potential. As deep learning models for drug synergy become more complex, causal alignment becomes increasingly vital to ensure comprehensive and reliable performance.

**Table 1:** Performance comparison of the three baseline models after embedding CADS across five tasks with five runs.

Score	Model	Validating				Testing			
		MAE	RMSE	*R* ^2^	PCC	MAE	RMSE	*R* ^2^	PCC
*Loewe*	DeepSynergy	6.236± 0.079	9.097± 0.095	0.624± 0.008	0.792± 0.005	6.224± 0.079	9.063± 0.100	0.623± 0.008	0.792± 0.006
	+ CADS	6.185 ± 0.035	9.047 ± 0.047	0.628 ± 0.004	0.796 ± 0.004	6.155 ± 0.033	8.997 ± 0.039	0.628 ± 0.003	0.796 ± 0.003
	DeepDDS	6.268± 0.056	9.130± 0.064	0.621± 0.005	0.791± 0.004	6.269± 0.063	9.156± 0.069	0.615± 0.006	0.787± 0.005
	+ CADS	6.200± 0.039	9.048± 0.039	0.628 ± 0.003	0.796 ± 0.002	6.196± 0.046	9.060± 0.050	0.623± 0.004	0.794± 0.002
	SynergyX	6.288± 0.115	9.240± 0.096	0.612± 0.008	0.787± 0.004	6.238± 0.106	9.195± 0.094	0.612± 0.008	0.787± 0.004
	+ CADS	**5.978** 0.069	**8.900** 0.077	**0.640** 0.006	**0.801** 0.004	**5.946** 0.082	**8.855** 0.087	**0.640** 0.007	**0.800** 0.004
*HSA*	DeepSynergy	3.998± 0.072	5.948± 0.086	0.787± 0.006	0.888± 0.004	3.955± 0.073	5.893± 0.085	0.790± 0.006	0.889± 0.003
	+ CADS	3.963 ± 0.065	5.907 ± 0.064	0.790 ± 0.005	0.889 ± 0.003	3.931± 0.072	5.867 ± 0.074	0.792 ± 0.005	0.890 ± 0.003
	DeepDDS	4.080± 0.027	6.059± 0.021	0.779± 0.002	0.883± 0.001	4.060± 0.029	6.050± 0.022	0.779± 0.002	0.883± 0.001
	+ CADS	3.985± 0.080	5.972± 0.079	0.786± 0.006	0.887± 0.003	3.971± 0.076	5.971± 0.076	0.785± 0.005	0.886± 0.003
	SynergyX	3.965 ± 0.149	6.058± 0.165	0.779± 0.012	0.887± 0.004	3.928± 0.136	6.029± 0.158	0.781± 0.011	0.888± 0.004
	+ CADS	**3.755** 0.021	**5.806** 0.026	**0.797** 0.002	**0.893** 0.001	**3.731** 0.028	**5.771** 0.038	**0.799** 0.003	**0.894** 0.001
*Bliss*	DeepSynergy	4.540± 0.137	6.949± 0.179	0.880± 0.006	0.938± 0.003	4.538± 0.139	7.059± 0.183	0.877± 0.006	0.937± 0.003
	+ CADS	4.466± 0.086	6.861± 0.102	0.883± 0.003	0.940± 0.002	4.472± 0.078	6.977± 0.098	0.880± 0.003	0.939± 0.002
	DeepDDS	4.698± 0.094	e7.116± 0.148	0.874± 0.005	0.935± 0.003	4.720± 0.104	7.269± 0.128	0.870± 0.005	0.933± 0.002
	+ CADS	4.480± 0.069	6.850± 0.069	0.883± 0.002	0.940± 0.001	4.491± 0.076	7.024± 0.094	0.879± 0.003	0.938± 0.002
	SynergyX	4.324 ± 0.140	6.786 ± 0.153	0.885 ± 0.005	0.942 ± 0.002	4.311 ± 0.138	6.932 ± 0.159	0.882 ± 0.005	0.940 ± 0.002
	+ CADS	**4.134** 0.029	**6.588** 0.039	**0.892** 0.001	**0.944** 0.001	**4.144** 0.021	**6.745** 0.014	**0.888** 0.000	**0.942** 0.000
*ZIP*	DeepSynergy	3.434± 0.057	5.244± 0.061	0.782± 0.005	0.885± 0.003	3.438± 0.059	5.380± 0.068	0.776± 0.006	0.881± 0.003
	+ CADS	3.371 ± 0.012	**5.174** 0.014	**0.788** 0.001	**0.888** 0.001	3.374 ± 0.013	**5.313** ± 0.019	**0.782** ± 0.002	**0.885** ± 0.001
	DeepDDS	3.576± 0.068	5.402± 0.076	0.769± 0.007	0.877± 0.004	3.585± 0.081	5.556± 0.087	0.762± 0.007	0.873± 0.004
	+ CADS	3.423± 0.041	5.253± 0.044	0.781± 0.004	0.884± 0.002	3.436± 0.049	5.444± 0.034	0.771± 0.003	0.879± 0.002
	SynergyX	3.409± 0.029	5.277± 0.041	0.779± 0.003	0.886± 0.001	3.411± 0.022	5.440± 0.028	0.771± 0.002	0.881± 0.001
	+ CADS	**3.339** 0.016	5.193 ± 0.018	0.786 ± 0.002	0.887 ± 0.001	**3.338** 0.008	5.354 ± 0.009	0.779 ± 0.001	0.883 ± 0.001
*S-Score*	DeepSynergy	6.134± 0.103	8.442 ± 0.111	0.761 ± 0.006	0.873± 0.004	6.187± 0.118	8.544± 0.126	0.758± 0.007	0.872± 0.004
	+ CADS	**6.058** ± 0.037	**8.375** ± 0.050	**0.765** ± 0.003	**0.875** ± 0.002	**6.095** ± 0.044	**8.452** ± 0.053	**0.763** ± 0.003	**0.874** ± 0.002
	DeepDDS	6.129± 0.034	8.468± 0.024	0.760± 0.001	0.873± 0.001	6.180± 0.026	8.563± 0.030	0.757± 0.002	0.871± 0.001
	+ CADS	6.107± 0.021	8.443± 0.033	0.761 ± 0.002	0.874 ± 0.001	6.144 ± 0.028	8.528 ± 0.034	0.759 ± 0.002	0.873 ± 0.001
	SynergyX	6.290± 0.037	8.710± 0.039	0.746± 0.002	0.868± 0.001	6.325± 0.028	8.801± 0.041	0.743± 0.002	0.867± 0.001
	+ CADS	6.086 ± 0.078	8.502± 0.089	0.758± 0.005	0.871± 0.002	6.158± 0.076	8.634± 0.084	0.753± 0.005	0.869± 0.002

**Bold** and underlined values indicate the best and second-best results in specific task, respectively. The colors **RED** and **PINK** denote statistically significant performance gains relative to the post-CADS embedding state, corresponding to *t*-test (Kim 2015) *P*-values below 0.01 and 0.05, respectively.

### 3.2 Causal genes ablation

To validate whether the model relies more heavily on causal genes during prediction, we conducted ablation experiments targeting both causal genes and trivial genes. We performed testing across five independent replicates of the CADS embedded within SynergyX (embedding five synergy metrics) by applying zero-masking to the top 1000 genes within the designated causal group and trivial group for each drug combination-cell line pair. As shown in Fig. 2, our analysis revealed that model performance is superior following ablation of the top trivial genes compared to ablation of the top causal genes. This finding indicates that masking causal gene information resulted in greater overall performance degradation, demonstrating that the model predominantly depends on causal gene information when generating predictions.

### 3.3 Robustness test

In this section, to analyse the robustness of CADS, we introduced few-shot learning and provided a demonstration of its application potential for novel drug combination discovery.

#### 3.3.1 Few-shot learning

To validate whether CADS can accurately infer cell-drug interactions under limited data scenarios, we employed a few-shot learning strategy, training the model using selected datasets [0.3, 0.5, 0.7] according to the method described in Appendix Section E.2.3, available as supplementary data at *Bioinformatics* online.

**Figure 3:**
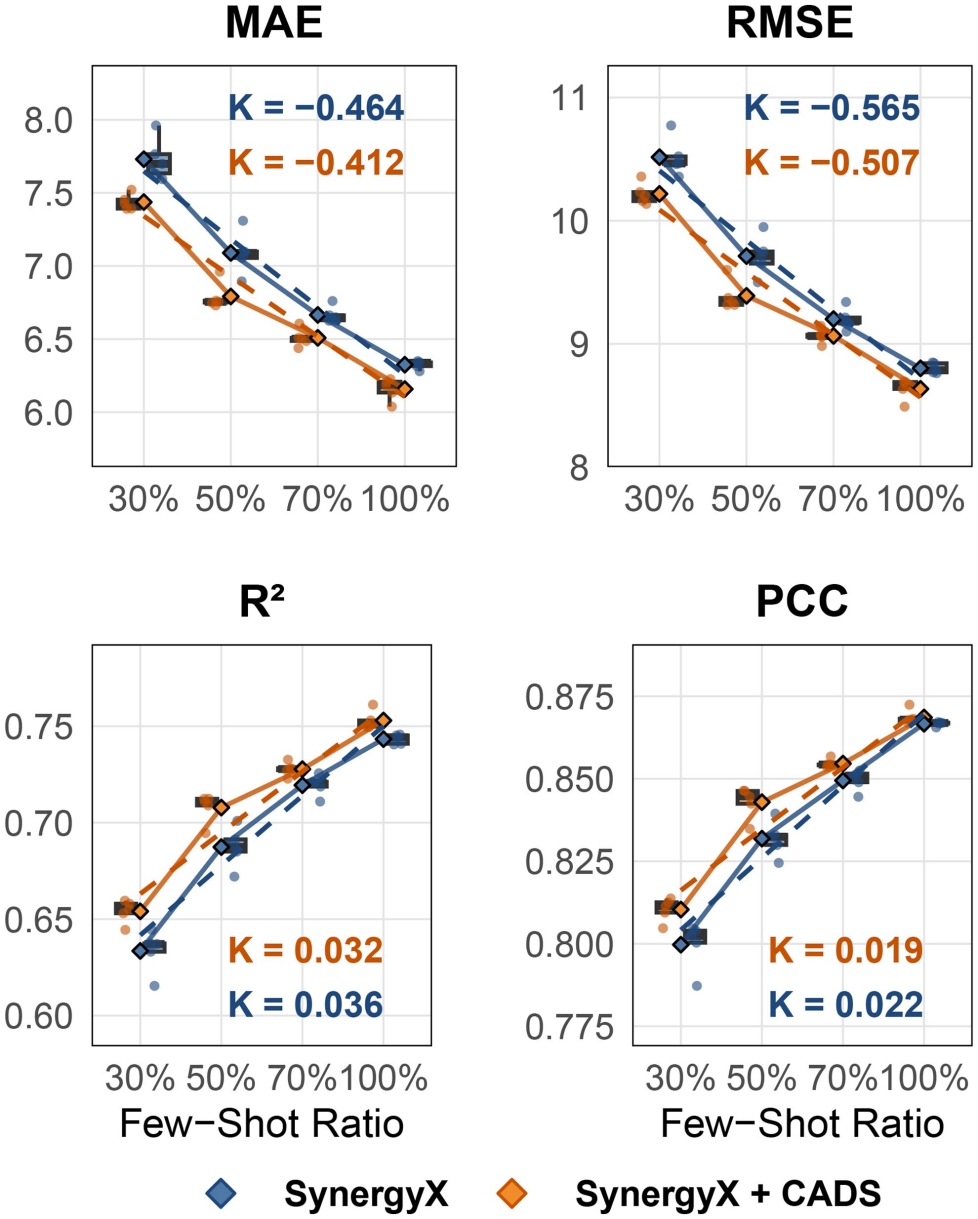
The *Paired Comparison Plot* illustrates four evaluation metrics from five sets of replicate experiments, comparing SynergyX+CADSand SynergyX under varying training set sizes for *S-Score* prediction.

To statistically quantify the benefit of the causal mechanism under data-scarce conditions, we conducted a linear regression analysis on the performance curves relative to the training set size. As illustrated in Fig. 3, we fitted the performance trends of both the baseline and the CADS-integrated models using linear functions (y=kx+b). The slope *k* serves as a critical indicator of the model’s sensitivity to data availability. A steeper slope implies a rapid degradation in performance as data decreases. Our analysis reveals that the absolute value of the slope for the CADS-integrated model ($\left|k_{\mathrm{CADS}}\right|$) is notably smaller than that of the baseline ($\left|k_{\mathrm{Base}}\right|$). This “flattened” trend line demonstrates that the CADS module effectively enhances the model’s robustness. It attenuates the rate of performance degradation when training samples are limited, thereby validating that the causal mechanism plays a vital role in maintaining predictive stability in few-shot learning tasks. Table 4, available as supplementary data at *Bioinformatics* online shows the few-shot learning results of the three baselines with CADS embedded.

#### 3.3.2 Novel drug combination discovery

To further investigate the robustness of CADS and its generalization capability for novel drug combinations, we assessed the capacity of CADS for both novel drug combination discovery and drug repurposing. Given the vast unexplored drug space, we selected several commonly used cancer cell lines, including HCT116 (Colon cancer), A549 (Lung cancer) and MDA-MB-231 (Breast cancer) according to the method described in Appendix Section E.2.4, available as supplementary data at *Bioinformatics* online. For drug combinations, we chose those present in the dataset but without documented experimental data paired with these cell lines. Through the aforementioned screening methodology, we identified a total of 20,338 novel drug combinations across the three cell lines. We utilized CADS trained on five independent replicated trials to predict the synergy of the aforementioned novel drug combinations. Table 2 presents the top four novel drug combinations, ranked by predicted S-Scores, across three distinct cell lines.

**Table 2:** Novel drug combinations.

Cell line	Tissue	Drug A	Drug B	Predicted S-Score	Supporting Publications	Year
HCT-116	Colon	BI-2536	MK-2206	63.44± 2.00	PMID: 27699933 (Nonomiya *et al.* 2016)	2016
		AZD6738	AZD7762	62.29± 5.20	PMID: 36230796 (Deng *et al.* 2022)	2022
		MK-1775	Vincristine	60.51± 5.37	PMID: 39464635 (Song *et al.* 2024)	2024
		Erlotinib	Vincristine	59.64± 5.08	PMID: 40427071 (Li *et al.* 2025)	2025
A549	Lung	BI-2536	Dasatinib	52.56± 5.46	PMID: 32520674 (Zhang *et al.* 2021)	2021
		RAF265	Selumetinib	50.15± 6.01	PMID: 25199829 (Lamba *et al.* 2014)	2013
		AZD2014	AZD3463	49.95± 3.64	–	–
		BI-2536	RAF265	49.65± 6.27	(Weekes and Hidalgo 2010)	2010
MDA-MB-231	Breast	Bortezomib	Fludarabine	47.55 ± 2.83	–	–
		Bortezomib	Navitoclax	46.92± 4.14	PMID: 31370269 (Lim *et al.* 2019)	2019
		Bortezomib	Erlotinib	46.84± 2.75	PMID: 24747441 (Seguin *et al.* 2014)	2014
		Bortezomib	Doxorubicin	45.22± 4.87	PMID: 21147690 (Irvin *et al.* 2010)	2010

The table presents the mean values, standard deviations, and relevant references for S-Score predictions of novel drug combinations, derived from five sets of replicate experiments using SynergyX+CADS.

To validate these predictions, we performed an extensive literature search. This revealed supporting evidence in the published literature for 10 out of the 12 entries (83.33%), thereby confirming the reliability of CADS in predicting novel drug combinations.

Notably, given that the DrugComb dataset used for training was updated in 2021 (Zheng *et al.* 2021), 40% confirmed novel drug combination entries were reported after this time point. This observation demonstrates the extensibility of the CADS, indicating its capability to discover novel drug combination-cell line causal relationships from a limited dataset.

### 3.4 Intepretability

In this section, we aimed to identify the key features of CADS that enable enhanced prediction accuracy.

#### 3.4.1 Cell embedding

**Figure 4:**
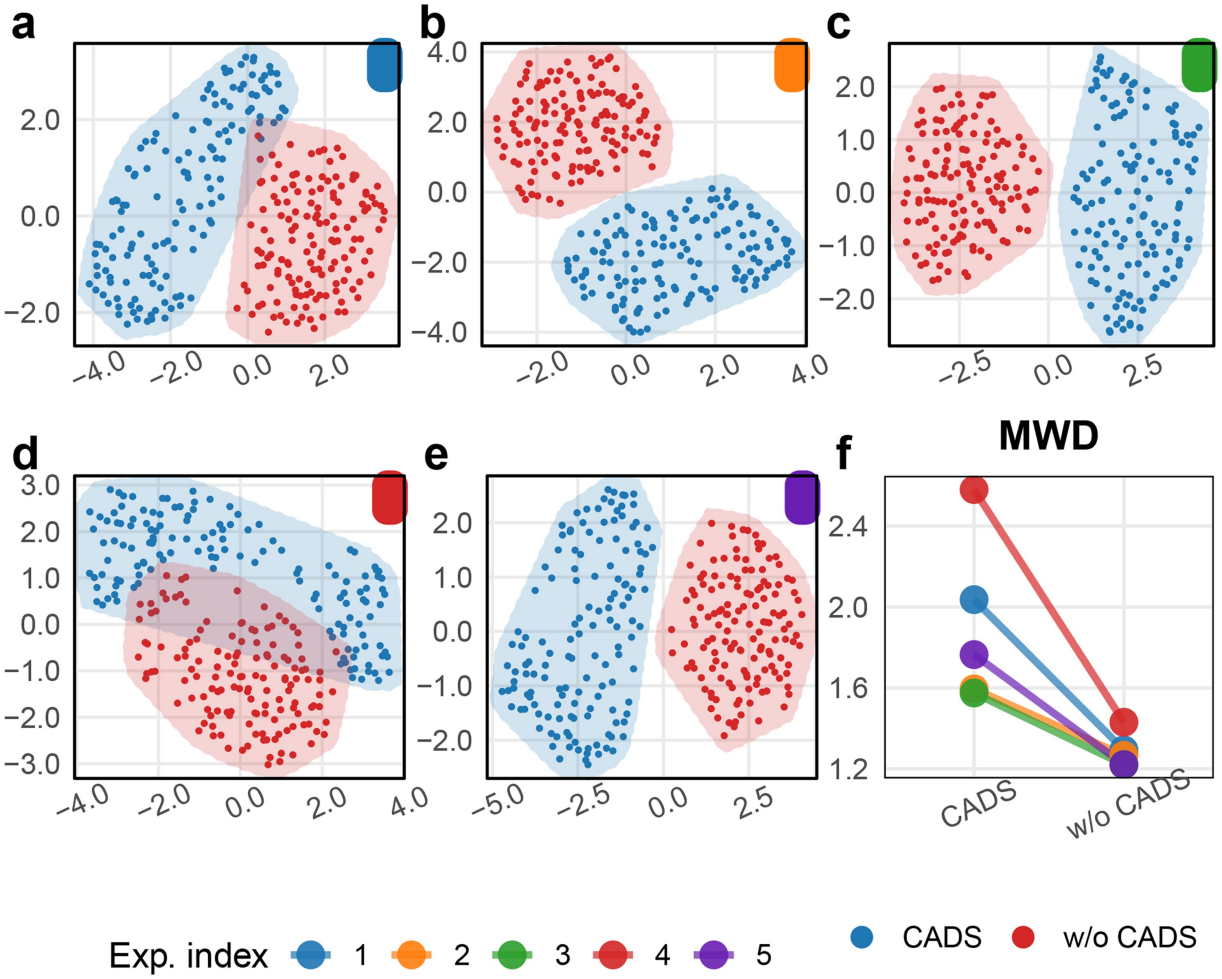
(a–e) The UMAP visualization illustrates the distribution of 141 cell line representation vectors from the causal component, derived from five sets of replicate experiments using SynergyX+CADS and SynergyX for *S-Score* prediction. (f) The Paired Comparison Plot illustrates the Mean Within-Class Distance (Schroff *et al*. 2015) across five groups of representation distributions.

Based on the critical importance of cell line characterization data for determining whether drug combinations exhibit synergistic effects on specific cell lines, we are particularly interested in whether integrating CADS can enhance the model’s capacity to capture cell line-specific features. In other words, we seek to investigate whether incorporating causal inference mechanisms enables the baseline models to generate more comprehensive representations of cell line information. To address this, we extracted the final-layer representational vectors of 141 cell lines from the five-independent experiment cell line encoder in SynergyX—both with and without CADS integration—at the stage immediately prior to drug combination-cell line interaction. Fig. 4A–E presents the UMAP visualizations comparative analysis derived from five-independent replicate experiments. Based on consistent parameter configurations per batch, as shown in Fig. 4F, we observed increased Mean Within-Class Distance (Schroff *et al.* 2015) in every replicate experiment following CADS integration, indicating that CADS enhances feature separability in cell line representations.

#### 3.4.2 General causal genes

**Figure 5:**
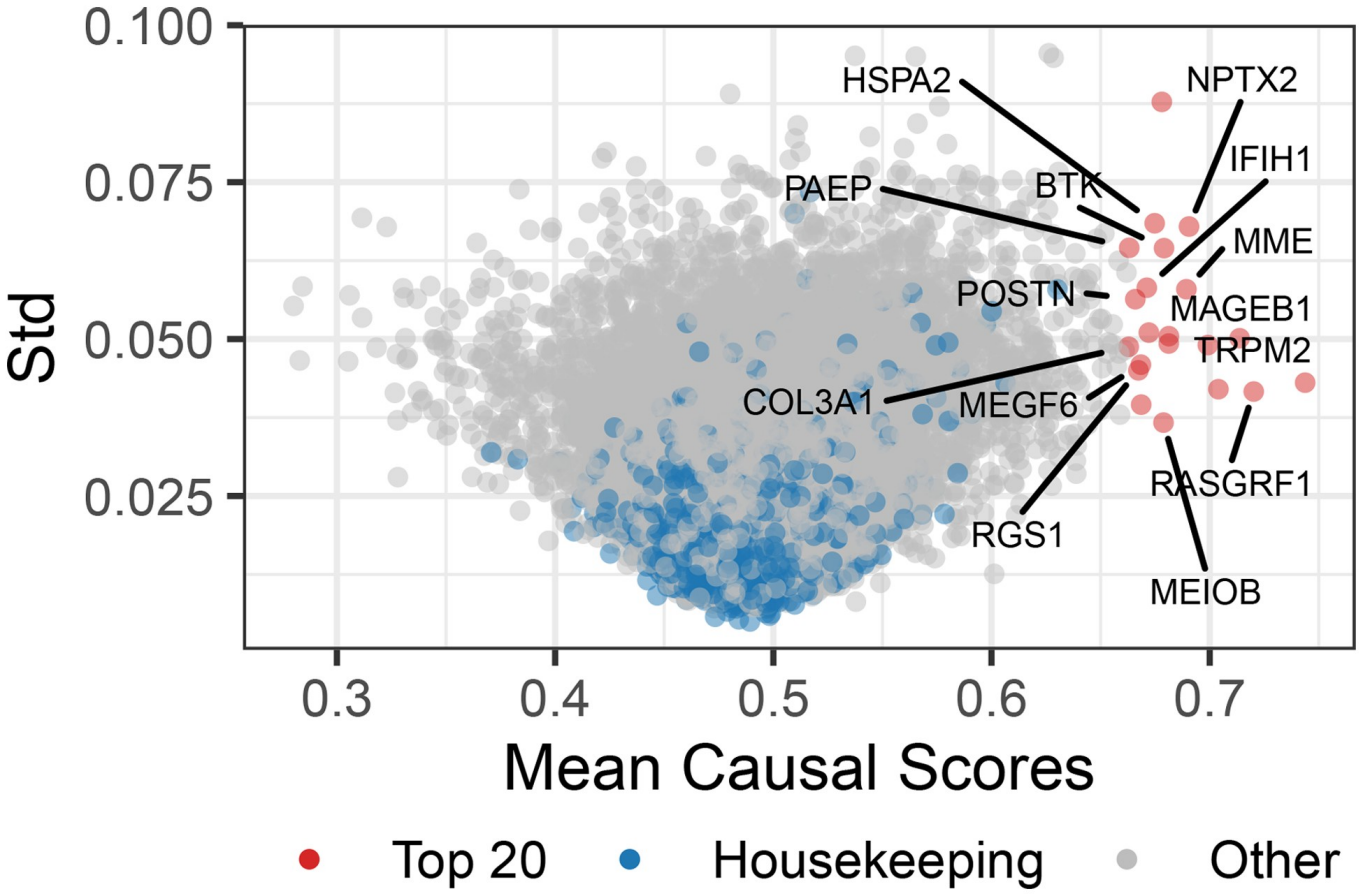
The volcano plot illustrates the mean and standard deviation of causal scores for 14,749 genes across 141 cell lines, derived from five sets of replicate experiments using SynergyX+CADS to predict *S-Score*.

To assess whether CADS isolates cell line-invariant causal information, we performed pan-cancer analysis of context-agnostic driver genes. Specifically, we computed the mean value and standard deviation of causal scores across 14 749 genes in a panel of 141 cell lines. Figure 5 displays the distribution of mean and variance for causal scores across genes. For comparative purposes, we adopted the housekeeping genes (Eisenberg and Levanon 2013) as the reference set. As illustrated, most housekeeping genes exhibit causal scores clustered between 0.45 and 0.55 with narrow variance, indicating that genes demonstrating low inter-cell-line variability, such as housekeeping genes, exert weaker effects on pharmacological synergy determination—a finding consistent with our initial hypothesis. We further investigated pan-causal genes in Appendix Section E.3.1 and E.3.2, available as supplementary data at *Bioinformatics* online.

#### 3.4.3 Validation of specific causal genes

**Figure 6:**
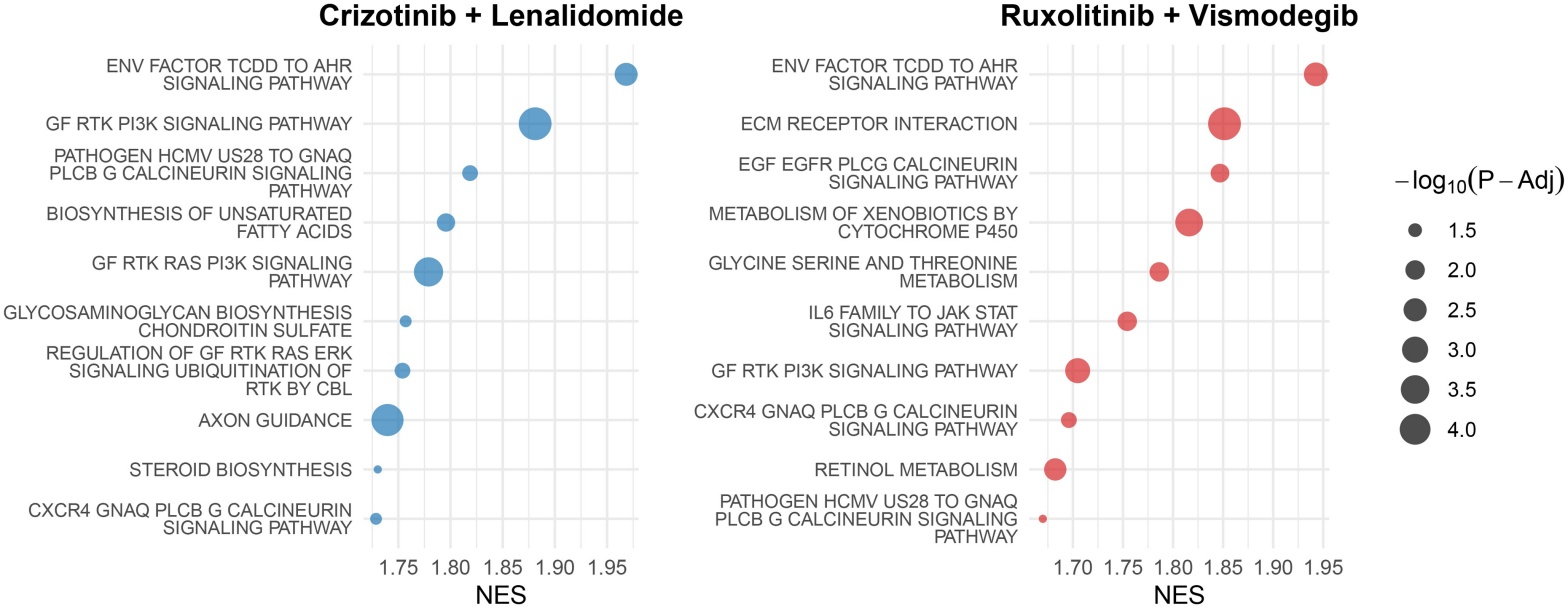
The bubble plot illustrates the top 10 enriched pathways identified through GSEA analysis of causal genes associated with two drug combinations, utilizing KEGG pathway sets (*n* = 369).

To validate the capability of CADSusing external knowledge bases, we assessed the homogeneity and heterogeneity of causal genes influencing distinct drug combinations. We selected the two drug combinations with the broadest cellular coverage: (*Ruxolitinib + Vismodegib*) (89 of 141 cell lines) and (*Crizotinib + Lenalidomide*) (87 of 141 cell lines). Subsequently, gene-level attribution scores for both drug combinations across distinct cell lines were computed using Integrated Gradients (Sundararajan *et al.* 2017). Mean contribution values were derived and subjected to Gene Set Enrichment Analysis (GSEA) (Korotkevich *et al.* 2016) in KEGG_LEGACY and KEGG_MEDICUS subsets (n = 369) in the MSigDB database. Figure 6 illustrates the top 10 enriched pathways based on the Normalized Enrichment Score (NES) for the two drug combinations.

Regarding the *Crizotinib + Lenalidomide* combination, *Lenalidomide* enhances immunoglobulin secretion in primary human B lymphocytes through activation of phosphatidylinositol 3-kinase δ (PI3K-δ)-dependent signaling cascades, a mechanism critical for its immunomodulatory effects in lymphoid malignancies (Lapalombella *et al.* 2010). *Crizotinib* exerts stronger antagonistic effects on receptor tyrosine kinase (RTK)-centric pathways, directly inhibiting ALK/MET-driven PI3K/RAS/ERK signal transduction to induce apoptosis and suppress proliferation in ALK-rearranged non-small cell lung cancer (NSCLC) cells (Shaw *et al.* 2013). Among the top 10 enriched pathways, RTK and PIK3 related pathways accounted for 30% (3/10), collectively demonstrating that CADS successfully captured core genetic determinants of this drug combination.

Regarding the *Ruxolitinib + Vismodegib* combination, several identified pathways demonstrate varying degrees of association with *Ruxolitinib’*s mechanism of action. Notably, the IL-6 family to JAK-STAT signaling pathway is directly implicated, as *Ruxolitinib* potently suppresses JAK-STAT activation downstream of IL-6 and related cytokines. The Hedgehog signaling pathway directly associated with *Vismodegib* exhibited a NES of 1.212 with an adjusted p-value > 0.05, indicating lack of statistical significance under conventional thresholds. This finding indicates that CADS successfully delineated core genetic determinants influencing *Ruxolitinib’*s mechanism, while partially underrepresenting *Vismodegib*-associated pathways, demonstrating a moderate feature selection bias in the model’s analytical framework. We further analyzed this phenomenon in the Appendix Section E.3.3, available as supplementary data at *Bioinformatics* online.

This comparative analysis of causal genes between the two drug combinations validates that CADS effectively discriminates combinatorial regulatory mechanisms by revealing heterogeneous drivers underlying convergent phenotypes, thereby establishing its novel capability to decipher context-specific synergistic logic rather than merely memorizing generalizable gene sets. The consistency between the model-identified pathways and the experimentally established mechanisms of action documented in external databases serves as a validation of CADS’s prediction reliability.

## 4 Conclusion

In this study, we introduced CADS, a novel framework for synergistic drug combination prediction. The model leverages causal inference principles to decouple biologically causal genes that critically influence drug response from trivial genetic associations within cell line profiles, thereby enhancing baseline predictive architectures. Experimental validation demonstrated concurrent improvements in both predictive accuracy and robustness against biological noise upon integration of the CADS module. Ablation studies confirmed CADS’s capability to accurately isolate mechanistically relevant causal features, while representation analysis revealed that refined cell line embeddings underlie its performance gains. Biological interpretability analyses elucidated CADS’s capacity to extract both pan-causal genes governing broad therapeutic responses and combination-specific mediators of synergistic effects.

Our analysis indicates that the “dominant-drug effect” is stochastic, with fluctuations in attention scores directly correlating to pathway sensitivity (e.g., in the Ruxolitinib-Vismodegib combination). Rather than enforcing a rigid symmetry, CADS supports a “contribution-aware” strategy: users can select specific experimental replicates based on drug contribution scores to optimize the identification of mechanisms of action.

Finally, we acknowledge potential threats to validity. Regarding internal validity, the high dimensionality and inherent noise in genomic data, coupled with the sparsity of available drug synergy labels, may pose challenges to the stability of causal discovery. Regarding external validity, our study relies on in vitro cell-line benchmarks; thus, generalizing these findings to in vivo clinical settings remains a challenge due to the complex tumor microenvironments that are not fully replicated in cell cultures.

Collectively, CADS establishes a paradigm for context-aware synergy prediction that transcends conventional feature correlation approaches, offering both computational efficacy and mechanistic interpretability for combinatorial drug discovery.

## Supplementary Material

btag010_Supplementary_Data

## Data Availability

The data and source code of CADS are available at: https://github.com/HuaiwuZhang/causalDC.
