# Supplementary material for: A causal inference framework for identifying essential genes to enhance drug synergy prediction
Source: Bioinformatics. 2026 May 28;42(5):btag010. doi: 10.1093/bioinformatics/btag010 (PMC13218379; doi:10.1093/bioinformatics/btag010)
Supplement: btag010_Supplementary_Data [file btag010_supplementary_data.pdf]

A. Notation

We standardize the following terms used throughout the paper:

- **Causal and trivial genes (G_c/G_t).** G_c is the subset of genes whose variation causally contributes to drug synergy for a given cellular context; G_t denotes the complementary subset that is non-causal (trivial) with respect to synergy in our setting. Unless stated otherwise, $G_c \cup G_t$ equals the modeled gene set and $G_c \cap G_t = \varnothing$. This separation is a modeling device to capture heterogeneity in feature relevance; instead of assuming full prior knowledge of G_c, it permits the development of a deep learning framework to isolate G_c and G_t from the complete set of genes.
- **Drug–cell representation (R_{dc}).** R_{dc} denotes the joint representation of a drug pair and a cell line, obtained by fusing drug features (e.g., SMILES, 2D molecular graph) with cell-line molecular features (e.g., expression, mutation, copynumber).
- **Backdoor.** We use "backdoor" to refer to Pearl's backdoor criterion: a set Z satisfies the backdoor criterion relative to $(X \rightarrow Y)$ if Z blocks all backdoor paths from X to Y and no element of Z is a descendant of X. In our context, we construct Z from gene subsets to adjust for confounding between drug–cell factors and synergy.
- **do-calculus.** We use the notation $\mathrm{do}(\cdot)$ to denote interventions and apply do-calculus rules to transform interventional distributions into observational ones when backdoor conditions are satisfied.
- **Interventional distribution (P_m).** We use $P_m(\cdot)$ to denote the interventional distribution resulting from a do-operation, distinguishing it from the observational distribution $P(\cdot)$.

B. Related Work

B.1. Deep causal learning

Traditional causal inference aims to investigate methods for learning a causal model applicable across diverse distributions; such a model encapsulates causal mechanisms and facilitates subsequent interventions or counterfactual inferences (Pearl et al., 2016). However, real-world observations frequently originate not from fundamental inference units—such as random variables connected within a causal graph—but rather from high-dimensional raw data (Schölkopf et al., 2021). Consequently, causal representation learning has emerged to integrate deep learning with causal inference, finding widespread application in fields such as computer vision (Lippe et al., 2022), recommender systems (Wang et al., 2023), graph (Sui et al., 2022) and spatio-temporal data mining (Xia et al., 2023; Luo et al., 2024). Similarly, within the biomedical domain, numerous methodologies have incorporated the principles of causal inference. For instance, (Liu et al., 2021) utilizes causal inference to simulate randomized clinical trials on real-world data, thereby effectively identifying drug repurposing candidates for coronary artery disease. Subsequently, CausalCell (Wen et al., 2023) applied causal mechanisms to single-cell downstream analysis. More recently, GRANGER (Chen et al., 2025) inferred gene regulatory networks from time-series data based on causal cyclic autoencoders. Nevertheless, no existing studies have applied causal inference specifically to drug synergy data. Therefore, in this study, we adopt a causal perspective to investigate cell line modeling and leverage causal techniques to identify key genes influencing drug combinations.

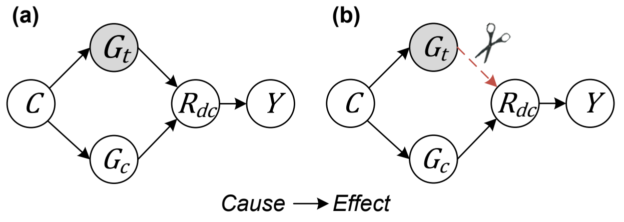

Fig. S1. SCMs of (a) drug synergy prediction; (b) back-door adjustment for trivial genes.

C. Preliminary

C.1. Structural causal model

As illustrated in (Zhang and Tang, 2025), we conducted a causal analysis of the drug combination response process modelling and constructed a structural causal model (SCM) (Pearl et al., 2000) in Figure S1a. The model reveals the causal relationships among five variables: cell line data C, causal gene G_c, trivial gene G_t, drug-cell representation R_{dc}, and drug synergy Y, where directed edges between variables represent causal relationships: cause → effect. The following is an introduction to the SCM:

- $\boldsymbol{G_c \leftarrow C \rightarrow G_t}$. The variable G_c represents causal genes in the cell line that directly influence drug efficacy, while G_t denotes other trivial genes. Since G_c and G_t naturally coexist in the cell line data C ($G_c \in C, G_t \in C$), these causal relationships are inherently established.
- $\boldsymbol{G_c \rightarrow R_{dc} \leftarrow G_t}$. The variable R_{dc} represents the joint representation of the cell line and drug information. Traditional learning strategies generate R_{dc} by simultaneously interacting causal genes (G_c) and trivial genes (G_t) with drug information.
- $\boldsymbol{R_{dc} \rightarrow Y}$. The ultimate goal of the joint representation R_{dc} is to predict the efficacy of drug combinations. Thus, the model performs a regression prediction for Y based on the representation R_{dc}.

Scrutinizing this SCM, we identify a backdoor path from G_c to Y, *i.e.*, $G_c \leftarrow C \rightarrow G_t \rightarrow R_{dc} \rightarrow Y$, where the trivial genes G_t acts as a confounder between G_c and Y. Even if no direct link exists between G_c and Y, this backdoor path induces a spurious correlation between them. For instance, the model may rely on G_t instead of G_c for predictions, leading to errors. Therefore, blocking the backdoor path and compelling the model to prioritize interactions between causal genes (G_c) and drug information is crucial.

C.2. Backdoor adjustment

The aforementioned illustration highlights that shielding regression models from the influence of trivial genes G_t is pivotal to fully leveraging causal genes G_c. Therefore, as shown in Figure S1b, we should eliminate backdoor paths to achieve joint representation of the cell line and drug information R_{dc}, rather than directly modelling the confounded $P(Y|G_c)$ influenced by trivial genes G_t (Sui et al., 2022). Manually distinguishing causal genes from trivial genes among tens of thousands of candidates is inherently challenging and

impractical. Fortunately, causal inference theory (Pearl et al., 2000; Pearl, 2014) offers a viable solution to address this challenge. By applying the do-calculus to causal genes G_c, we eliminate backdoor paths through estimating $P_m(Y|G_c) = P(Y|do(G_c))$. To achieve causal identifiability, it is necessary to stratify the trivial genes G_t into three distinct layers between G_c and Y:

- **Marginal invariance of trivial genes**: The marginal distribution $P(G_t = g)$ remains invariant under the intervention $do(G_c \perp\!\!\!\perp G_t)$, since the intervention exclusively severs backdoor pathways while preserving the intrinsic distribution of trivial genes, where $\perp\!\!\!\perp$ represents conditional independence. Formally:

$$P(G_t = g) = P_m(G_t = g)$$

- **Structural invariance of response mechanism**: The conditional distribution $P(Y|G_c = c, G_t = g)$ maintains its structural form under intervention, as the response mechanism $Y|G_c, G_t$ operates independently of the G_c-G_t causal relationship. This yields:

$$P_m(Y|G_c = c, G_t = g) = P(Y|G_c = c, G_t = g)$$

- **Orthogonality under intervention**: The causal intervention induces statistical independence between G_c and G_t by design, satisfying:

$$P_m(G_t = g|G_c = c) = P_m(G_t = g)$$

This orthogonalization constitutes the fundamental property of the **do-calculus** in our causal framework. Now we have:

$$\begin{aligned} P(Y \mid \mathrm{do}(G_c)) &= P_m(Y \mid G_c) \\ &= \sum\nolimits_{g \in \mathcal{V}(G_t)} P_m(Y \mid G_c, g), P_m(g \mid G_c) \\ &= \sum\nolimits_{g \in \mathcal{V}(G_t)} P_m(Y \mid G_c, g), P_m(g) \\ &= \sum\nolimits_{g \in \mathcal{V}(G_t)} P(Y \mid G_c, g), P(g), \end{aligned} \tag{1}$$

where $\mathcal{V}(G_t)$ denotes the value set of the variable G_t. At this stage, we have achieved backdoor adjustment.

C.3. Problem formulation

We consider the task of drug synergy prediction, where the goal is to estimate the synergistic effect of a drug pair on a given cell line. Let $d_A \in \mathcal{D}$ and $d_B \in \mathcal{D}$ denote two drugs, and let $c \in \mathcal{C}$ denote a cell line. Each drug is associated with a feature representation (e.g., 2D chemical structure, SMILES sequence), denoted by $\mathbf{x}_{d_A} \in \mathbb{R}^p$ and $\mathbf{x}_{d_B} \in \mathbb{R}^p$. Each cell line is associated with a feature representation (e.g., gene expression, mutation, copynumber), denoted by $\mathbf{x}_c \in \mathbb{R}^q$.

Given a training set

$$\mathcal{S} = \{(d_A^{(i)}, d_B^{(i)}, c^{(i)}, y^{(i)})\}_{i=1}^N,$$

where $y^{(i)} \in \mathbb{R}$ is an observed synergy score (e.g., Bliss, Loewe, HSA, ZIP, S-Score), the goal is to learn

$$\hat{y} = f_\theta(\mathbf{x}_{d_A}, \mathbf{x}_{d_B}, \mathbf{x}_c),$$

where f_θ is a deep learning model. Parameters are learned by minimizing MSE:

$$\min_\theta \frac{1}{N} \sum_{i=1}^N (f_\theta(\mathbf{x}_{d_A^{(i)}}, \mathbf{x}_{d_B^{(i)}}, \mathbf{x}_{c^{(i)}}) - y^{(i)})^2.$$

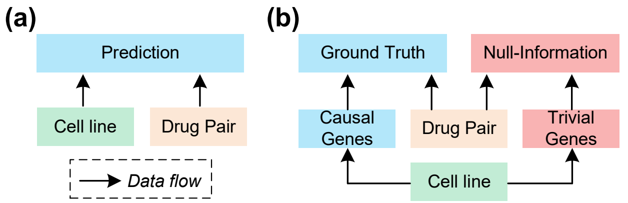

Fig. S2. Simplified data flow illustrating the causal mechanism within CADS. **(a)** *Base Phase:* The standard prediction workflow where the complete cell line profile and drug pair features are combined to generate the final synergy prediction. **(b)** *Causal Phase:* The standard prediction workflow where the complete cell line profile and drug pair features are combined to generate the final synergy common causal prediction.

Table S1. Computational cost analysis: Comparison of training time (in seconds) between baseline models and CADS -integrated variants on the S-Score dataset.

Method	DeepSynergy	DeepDDS	SynergyX
Base	346.5	465.3	493.0
+ CADS	389.4	506.6	994.1

Table S2. Model complexity analysis: Comparison of the number of parameters (in Millions) for baselines and CADS -integrated models.

Method	DeepSynergy	DeepDDS	SynergyX
Base	82.3	136.1	165.3
+ CADS	141.9	195.7	352.1

D. Methodology

D.1. Causal logic and simplified data flow

To assist in interpreting the model's design philosophy, we provide a simplified schematic of the data flow in Figure S2.

As shown in Figure S2a, during the baseline stage, the model takes the drug pair and the cell line profile as inputs to output a prediction score, similar to traditional deep learning approaches. However, the uniqueness of CADS lies in its training mechanism (Figure S2b). We hypothesize that not all genes in a cell line profile contribute to the drug response; some features are "trivial" or confounders that may lead to overfitting.

Therefore, CADS explicitly decouples the cell line features into two streams:

- **Causal Genes**: These features are learned to maximize the prediction accuracy against the Ground Truth.
- **Trivial Genes**: These features are forced to align with Null-Information (random noise or zero-synergy labels), effectively penalizing the model for relying on irrelevant data.

This simplified flow illustrates how CADS filters out noise to focus on the true causal mechanism of drug synergy.

D.2. Module complexity

To further evaluate the efficiency of the proposed method, we analyzed the computational complexity of the baseline models (DeepSynergy, DeepDDS, and SynergyX) before and after integrating the CADS module. We focused on two key metrics: training time (in seconds) and model size (number of

parameters in millions). The detailed comparisons are provided in Table S1 and Table S2 in the Appendix.

As observed in Table S1, the inclusion of CADS results in an increase in training time across all methods. For instance, the training time for SynergyX increases from 493.0 seconds to 994.1 seconds. Similarly, Table S2 shows that the number of parameters grows with the integration of CADS (e.g., from 165.3M to 352.1M for SynergyX). This increase in computational cost and model size is expected, as CADS introduces additional learnable components to better capture. Despite the higher complexity, the substantial performance gains achieved by CADS justify the additional computational resources required.

E. Experiments & Results

We conducted a series of experiments to validate the performance of our CADS. These experiments are designed to address the following Research Questions (RQs):

- **RQ1**: Does CADS really help improve drug synergy prediction performance?
- **RQ2**: Are the CADS predictions grounded in the identified causal genetic information?
- **RQ3**: How robust is CADS under varying conditions?
- **RQ4**: How to explain the predicted causal genes in biological context?

E.1. Experiments setting

E.1.1. Baselines & Evaluation metrics

To demonstrate the performance of our CADS, we experimentally integrated it into multiple deep learning architectures for predicting drug combination responses, including DeepSynergy (Preuer et al., 2018), DeepDDS (Wang et al., 2022) and SynergyX (Guo et al., 2024). We used four metrics to measure the performance, including Mean Absolute Error (MAE), Root Mean Square Error (RMSE), R-squared (R^2), and Pearson Correlation Coefficient (PCC). To ensure experimental reproducibility, we partitioned the data into training, validation, and test sets with an 8:1:1 ratio using random seed 42. Subsequently, we conducted five independent trials for each score, and the mean value of each score is reported.

E.1.2. Implementation details & Hyperparameters

Our model uses the Adam optimizer with the initial learning rate set to 0.0001, reduced by 0.5 every 100 epochs. To avoid overfitting, we employ an early stopping mechanism with a patience of 25 epochs. The batch size and embedding dimension were configured at 32 and 128, respectively. Gradient clipping is employed to mitigate the risk of exploding gradients during backpropagation, with the L2-norm of all trainable parameters' gradient vectors constrained to a maximum threshold of 5.0.

E.2. Dataset analysis

E.2.1. Dataset description

We used the same data as (Zhang and Tang, 2025). The input data utilized in our framework is categorized into three major types, including 1) drug synergy, 2) drug information, and 3) cell line information.

Drug synergy. We retrieved a total of 739,964 entries of drug combination synergy data on cancer cell lines from the DrugComb dataset (Zagidullin et al., 2019). Each entry consists of a drug pair, a cell line, and five synergy scores corresponding to different reference models (*S-Score*, *HSA*, *Bliss*, *Loewe*, and *ZIP*).

Drug information. The DrugComb database provides SMILES strings (Gaulton et al., 2012) for all individual drugs involved in the combinations. To conform to the input format specifications of our selected baseline methods, we transform the SMILES representations into distinct structural formats: sequence structures for MSA input and graph structures for GCN input:

- **Sequence**: We first converted them to canonical SMILES using the RDKit Python package, and then transformed them into Explainable Substructure Partition Fingerprint (ESPFs) (Huang et al., 2019). This approach enables the decomposition of SMILES into interpretable chemical substructures, which were also used in previous studies (Guo et al., 2024). To ensure uniform input dimensions, we padded the fingerprints to a maximum length of 150, filling shorter sequences with zeros. The final drug representation was constructed by integrating the drug substructure vectors with positional encodings derived from the Transformer architecture (Vaswani et al., 2017), followed by layer normalization and stochastic dropout operations. The resulting drug representation can be expressed as $\mathbf{D} \in \mathbb{R}^{L \times C}$, where $L = 150$ indicates the maximum length of drug substructures, and $C = 128$ represents the dimensionality of substructure embeddings.
- **2D graph**: Firstly, we also converted them to canonical SMILES. Next, we utilized the RDKit package to convert SMILES strings into graph structures, omitting hydrogen atoms during this process. All drug compounds collectively encompass 32 unique atom types. Atomic features are initially mapped to integer indices and subsequently learned through trainable embedding vectors updated during model optimization. Furthermore, edge structures exclusively represent covalent bonds between atoms without additional edge attributes.

For a more detailed description, please refer to Algorithm 1.

Cell line information. We retrieved gene-level features associated with cancer cell lines from the DepMap database (Ghandi et al., 2019), including copy number variation, gene expression, mutation, and RNAi-based gene dependency. The resulting cell line data can be represented as $\mathbf{C} \in \mathbb{R}^{F \times N}$, where F denotes the number of gene feature types, and N represents the total number of genes.

Data processing. We identified 14,749 genes that contain all the four feature types. Drug synergy data were then rigorously screened using a dual-phase protocol: (1) exclusion of drug pairs lacking canonical SMILES representations to ensure chemical standardization, followed by (2) elimination of low-frequency drugs that appear in fewer than five unique pairs. The drug synergy *S-Scores* underwent stringent quality control via the 3σ principle (Pukelsheim, 1994) to remove distributional outliers, followed by normalization. Finally, a hierarchical integration of the processed genomic and pharmacological datasets resulted in 307,564 drug-cell line associations, encompassing 398 compounds and 141 cancer cell lines with gene-level features. As shown in Table S3, while the initial cleaning step significantly refined the dataset structure, the subsequent 3σ outlier removal was highly targeted. It removed only 2,677 combinations, corresponding to a removal rate of approximately 0.86%. This

Table S3. Dataset statistics across different processing stages. The table reports the counts of drug-cell line combinations, unique drugs, and cell lines. The 3σ filtering step resulted in a data removal rate of 0.86%.

Stage	Combinations	Drugs	Cell Lines
Pre-cleaning	779,964	4,268	288
Post-cleaning	310,241	402	141
Post-3σ	307,564	398	141

indicates that the 3σ filtering effectively eliminated noise while preserving the vast majority of valid biological data.

Algorithm 1 Mathematical Representation of Drug Input Processing

Require: DrugComb dataset $\mathcal{D} = \{(d_i, d_j, c, \mathbf{s})\}$ where $d_i, d_j \in \mathcal{D}_{\text{drugs}}$, c denotes cell line, and $\mathbf{s} \in \mathbb{R}^5$ is the synergy score vector.

Ensure: Processed dataset

$\mathcal{D}^* = \left\{\left(\mathbf{D}_i^{\text{seq}}, \mathbf{D}_j^{\text{seq}}, \mathbf{D}_i^{\text{graph}}, \mathbf{D}_j^{\text{graph}}, c, \mathbf{s}\right)\right\}$.

Let $\mathcal{R}$ be the RDKit function for canonicalization and molecule construction.

Let $\mathcal{E}$: SMILES $\to \mathbb{Z}^+$ be the ESPF function that decomposes a SMILES string into a sequence of substructure indices.

for each unique drug d in $\mathcal{D}_{\text{drugs}}$ **do**

  $\text{smiles}_{\text{canon}} \leftarrow \mathcal{R}_{\text{canon}}(d.\text{smiles})$ ▷ Canonical SMILES

  Sequence representation:

  $\mathbf{f} = [f_1, f_2, \ldots, f_k] = \mathcal{E}(\text{smiles}_{\text{canon}})$ where $f_i \in \{1, 2, \ldots, N_{\text{sub}}\}$

  Pad $\mathbf{f}$ to fixed length $L = 150$: $\tilde{\mathbf{f}} = [f_1, \ldots, f_k, 0, \ldots, 0] \in \mathbb{Z}^{150}$

  Create a mask $\mathbf{m} \in \{0,1\}^{150}$: $m_i = \mathbb{I}[i \leq k]$

  $\mathbf{X} = \mathbf{W}_e \tilde{\mathbf{f}}$ ▷ Embedding: $\mathbf{W}_e \in \mathbb{R}^{128 \times N_{\text{sub}}}$, so $\mathbf{X} \in \mathbb{R}^{150 \times 128}$

  $\mathbf{P} \in \mathbb{R}^{150 \times 128}$: $P_{i,2j} = \sin(i/10000^{2j/128})$, $P_{i,2j+1} = \cos(i/10000^{2j/128})$

  $\mathbf{Z} = \mathbf{X} + \mathbf{P}$ ▷ Add positional encoding

  $\mathbf{Z}' = \text{LayerNorm}(\mathbf{Z})$ ▷ Layer normalization

  $\mathbf{D}^{\text{seq}} \leftarrow \text{Dropout}(\mathbf{Z}', p = 0.1)$

  Graph representation:

  $\mathcal{G} = (\mathcal{V}, \mathcal{E}) \leftarrow \mathcal{R}_{\text{mol}}(\text{smiles}_{\text{canon}})$ ▷ Molecular graph without hydrogens

  Let $n = |\mathcal{V}|$

  $\mathbf{v} = [v_1, v_2, \ldots, v_n]^\top$ where $v_i = \phi(\text{atom}_i) \in \{1, 2, \ldots, 32\}$ ▷ Atom type mapping

  $\mathbf{X}_v = \mathbf{W}_a \mathbf{v}$ ▷ Atom embedding: $\mathbf{W}_a \in \mathbb{R}^{d_g \times 32}$, so $\mathbf{X}_v \in \mathbb{R}^{n \times d_g}$

  $\mathbf{A} \in \{0,1\}^{n \times n}$: $A_{ij} = 1$ if bond between atom i and j exists, else 0

  $\mathbf{D}^{\text{graph}} = (\mathbf{X}_v, \mathbf{A}, n)$ ▷ Node features, adjacency matrix, and number of nodes

end for

Assemble $\mathcal{D}^*$ by replacing each drug in the original pairs with their $\mathbf{D}^{\text{seq}}$ and $\mathbf{D}^{\text{graph}}$ representations.

  return $\mathcal{D}^*$

E.2.2. Metrics correlation

As shown in Figure S3, Bliss and ZIP share a no-interaction null, yielding a high correlation. HSA and S-Score both measure

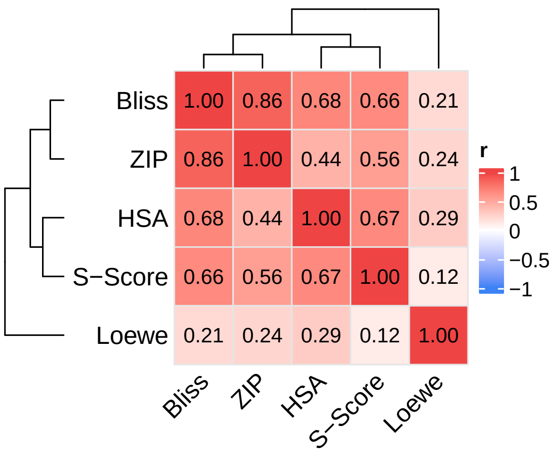

Fig. S3. Heatmap displays the Pearson correlation between five synergy scoring models: Bliss, ZIP, HSA, S-Score, and Loewe.

excess over the best single agent and therefore correlate strongly. Cross-family correlations (Bliss/ZIP vs. HSA/S-Score) are moderate because the baselines differ. Loewe, based on dose additivity and equivalent-dose isoboles, is conceptually distinct and sensitive to single-agent curve shapes, so it correlates weakly with the other metrics. The empirical pattern is consistent with these underlying null hypotheses (Ianevski et al., 2020).

E.2.3. Few-shot data split

To rigorously evaluate the generalization capability of CADS under data-scarce conditions, we implemented a deterministic data partitioning strategy to control randomness. We shuffled the entire training dataset using a fixed random seed (set to 42) to guarantee determinism. Subsequently, we constructed the few-shot subsets by sequentially selecting the first 30%, 50%, and 70% of the shuffled samples. This approach ensures that the subsets are nested (i.e., the 30% subset is strictly contained within the 50% subset). Crucially, these identical data splits were shared across all comparative methods (including baselines and CADS). This protocol eliminates performance variance arising from random data selection, thereby ensuring a fair comparison and full reproducibility of the experimental results.

E.2.4. Novel drug discovery dataset

Our dataset comprises 11,857 unique drug pairs. The three model cell lines are paired with specific subsets of these pairs where experimental data is available (A549: 4,903; HCT116: 5,438; MDA-MB-231: 4,892). To ensure rigorous evaluation in the novel prediction stage, we defined the prediction space as the strict complement of the labeled dataset. Specifically, we generated the full Cartesian product of drug pairs and cell lines ($3 \times 11,857$) and excluded any drug-pair–cell-line tuple that appeared in the training data (i.e., any tuple with available labels). We conducted inference solely on the remaining 22,338 unseen tuples ($35,571 - 15,233$). This exclusion procedure guarantees that the set of tuples used for novel prediction has zero intersection with the dataset used for model training, thereby eliminating any hidden overlap.

Table S4. Few-shot learning performance comparison of the three baseline models after embedding CADS in S-Score with five runs. Base and +CADS correspond to the model configurations without and with the CADS module embedded, respectively. Arrows (↓) and (↑) denote the performance improvement (%) after CADS embedding. The colors **RED** and **PINK** denote statistically significant performance gains relative to the post-CADS embedding state, corresponding to t-test (Kim, 2015) p-values below 0.01 and 0.05, respectively.

Model	Ratio	MAE			RMSE			R^2			PCC		
		Base	+ CADS	↓	Base	+ CADS	↓	Base	+ CADS	↑	Base	+ CADS	↑
DeepSynergy	1.0	6.187±0.118	6.095±0.044	1.490	8.544±0.126	8.452±0.053	1.071	0.758±0.007	0.763±0.003	0.684	0.872±0.004	0.874±0.002	0.333
	0.7	6.557±0.059	6.493±0.105	0.981	9.018±0.037	8.974±0.106	0.481	0.731±0.002	0.733±0.006	0.351	0.856±0.001	0.857±0.004	0.184
	0.5	6.886±0.055	6.752±0.050	**1.943**	9.433±0.054	9.311±0.057	**1.290**	0.705±0.003	0.713±0.004	1.072	0.840±0.002	0.846±0.002	**0.626**
	0.3	7.432±0.113	7.291±0.061	1.897	10.099±0.084	9.986±0.058	1.118	0.662±0.006	0.670±0.004	**1.137**	0.815±0.004	0.820±0.002	0.616
DeepDDS	1.0	6.180±0.026	6.144±0.028	0.576	8.563±0.03	8.528±0.034	0.406	0.757±0.002	0.759±0.002	0.260	0.871±0.001	0.873±0.001	0.151
	0.7	6.504±0.050	6.443±0.066	0.929	8.982±0.052	8.913±0.06	0.774	0.733±0.003	0.737±0.004	0.562	0.857±0.002	0.860±0.002	0.283
	0.5	6.814±0.031	6.773±0.074	0.597	9.359±0.026	9.313±0.102	0.494	0.710±0.002	0.713±0.006	0.399	0.844±0.001	0.845±0.845	0.208
	0.3	7.433±0.070	7.328±0.030	**1.417**	10.114±0.078	10.009±0.040	**1.040**	0.661±0.005	0.668±0.003	**1.063**	0.815±0.003	0.819±0.001	**0.512**
SynergyX	1.0	6.325±0.028	6.158±0.076	2.636	8.801±0.041	8.634±0.084	1.895	0.743±0.002	0.753±0.005	1.294	0.867±0.001	0.869±0.002	0.221
	0.7	6.665±0.055	6.508±0.061	2.355	9.201±0.088	9.064±0.058	1.489	0.719±0.005	0.728±0.003	1.154	0.849±0.003	0.855±0.001	0.605
	0.5	7.089±0.148	6.791±0.095	**4.202**	9.712±0.163	9.39±0.120	**3.324**	0.687±0.010	0.708±0.008	2.977	0.832±0.005	0.843±0.005	1.329
	0.3	7.731±0.144	7.437±0.054	3.796	10.516±0.156	10.218±0.087	2.835	0.634±0.011	0.654±0.006	**3.240**	0.800±0.007	0.810±0.004	**1.332**

E.3. Results

E.3.1. General causal genes

We further investigated pan-causal genes exhibiting elevated mean scores. Literature mining of the top 20 genes ranked by causal score mean revealed that 14 genes (70%) were functionally characterized in ≥ 3 tumor types and experimentally implicated in cancer pathogenesis and therapeutic response. This gene set encompasses both extensively characterized oncogenes (*i.e.,* TRPM2/BTK/POSTN) and novel cancer-associated genes identified through emerging research paradigms, such as cancer-testis (CT) genes (Wang et al., 2016) in recent years (*i.e.,* MEIOB). This discovery validates the capacity of CADS to capture pan-genomic determinants influencing drug synergy. Detailed in Table S5, these top-ranked candidates—selected based on mean causal scores across 141 cell lines—demonstrate robust literature support (S.P.), further confirming the reliability of the identified causal features. Beyond biological relevance, we assessed the clinical translatability of these findings by mapping the identified targets to approved pharmaceutical agents. As presented in Table S6, several high-scoring genes are recognized targets for FDA-approved drugs; for instance, TRPM2, BTK, and RASGRF1 are targeted by agents such as Paclitaxel (DB01229), Ibrutinib (DB09053), and Sunitinib (DB01268), respectively. The alignment between our model's output and existing pharmacopoeia—specifically the identification of targets for widely used chemotherapeutics like Cisplatin (COL3A1) and targeted therapies like Bevacizumab (POSTN)—highlights the potential of CADS to uncover actionable candidates for drug repurposing and combination therapy design.

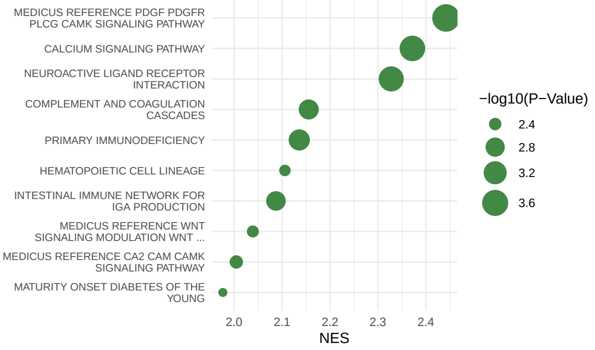

Fig. S4. The bubble plot illustrates the top 10 enriched pathways identified through GSEA analysis of pan-cancer causal genes, utilizing KEGG pathway sets (n = 369).

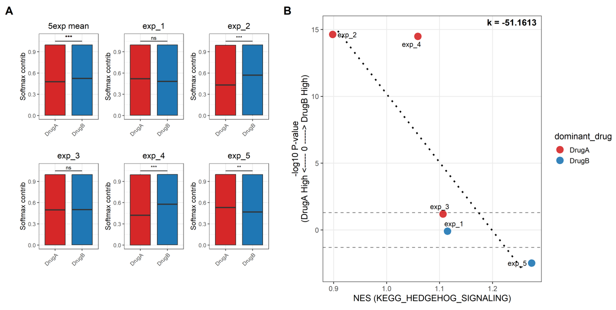

Fig. S5. **(A)** Boxplots showing the distribution of Softmax contribution scores for Drug A and Drug B across five independent experiments (exp_1 to exp_5) and their aggregated mean. The statistical significance was calculated using the t-test (ns: not significant, **: p < 0.01, ***: p < 0.001). The results highlight that drug dominance is stochastic across runs. **(B)** Scatter plot illustrating the correlation between the dominance significance ($-log_10P - value$ of Drug A vs. Drug B contribution) and the Normalized Enrichment Score (NES) of the KEGG_HEDGEHOG_SIGNALING pathway for the Ruxolitinib + Vismodegib combination. The dashed line represents the fitted linear regression ($k = -51.1613$), suggesting that the identification of drug-specific pathways is dependent on the model's attention distribution in that specific run.

E.3.2. Pan-cancer GSEA

As shown in Figure S4, our GSEA of the *pan-cancer* gene set shows a clear and connected pattern that fits well with known *pan-cancer* biology. The PDGF/PDGFR–PLCγ–Ca^{2+}–CaMK axis and the wider calcium pathway are both enriched, pointing to a continuous signal from RTKs to CaMK that appears across many cancer types. These pathways are linked to cell growth, movement, and blood vessel formation, and they have proven drug targets such as PDGFR inhibitors (Andrae et al., 2008; Demoulin and Essaghir, 2014). Signals from neuroactive

Table S5. The top 20 genes ranked by the mean causal scores across 141 cell lines, derived from five sets of replicate experiments using SynergyX+CADS to predict *S-Score*. Supporting Publications represent research papers or review articles directly focused on the gene in relation to 3 different tissues. * indicates that the number of relevant publications is fewer than 3. S.P. represents Supporting Publications.

**Rank**	**Gene**	**S.P.**
(1)	TRPM2	(Miller, 2019)(Ali et al., 2023)(Zeng et al., 2010)
(2)	RASGRF1	(Hunihan et al., 2022)(Chen et al., 2018)(Cooper et al., 2020)
(3)	TIGD7	*
(4)	C14orf132	*
(5)	NPTX2	(Xu et al., 2019)(Zhang et al., 2012)(Han et al., 2021)
(6)	MAMLD1	*
(7)	MME	(Zhu et al., 2024)(Li et al., 2019)(Wang et al., 2024)
(8)	CCL4L2	*
(9)	BTK	(Rada et al., 2018)(Guo et al., 2014)(Messex and Liou, 2021)
(10)	MEIOB	(Wang et al., 2016)(Gu et al., 2021)(Shiohama et al., 2014)
(11)	COL9A2	*
(12)	HSPA2	(Sojka et al., 2019)(Zhai et al., 2021)(Sojka et al., 2021)
(13)	MAGEB1	(Li et al., 2015)(Jia et al., 2019)(Li et al., 2020)
(14)	IFIH1	(Li et al., 2024)(Shi et al., 2024)(Yu et al., 2022)
(15)	GPR85	*
(16)	MEGF6	(Hu et al., 2018)(Qiang et al., 2025)(Mou et al., 2021)
(17)	RGS1	(Zhang et al., 2022)(Roh et al., 2017)(Sethakorn and Dulin, 2013)
(18)	POSTN	(Yu et al., 2021)(Oh et al., 2017)(Lu et al., 2023)
(19)	PAEP	(Tang et al., 2024)(Buenaventura et al., 2024)(Yang et al., 2026)
(20)	COL3A1	(Yang et al., 2022)(Zhang et al., 2020)(Zhang et al., 2021)

Table S6. The top 20 genes ranked by the mean causal scores across 141 cell lines, derived from five sets of replicate experiments using SynergyX+CADS to predict *S-Score*. The table displays drugs targeting these 20 genes (if more than three drugs are known, only three are shown. S.P. represents Supporting Publications.

**Target**	**Drug**	**DrugBank ID**	**S.P.**
TRPM2	Curcumin<br>Docetaxel<br>Paclitaxel	DB11672<br>DB01248<br>DB01229	(Ji et al., 2022)
RASGRF1	Sunitinib<br>Trametinib	DB01268<br>DB08911	(Cooper et al., 2020)<br>(Hunihan et al., 2022)
MME	Thiorphan	DB08626	(Mina et al., 2022)
BTK	Ibrutinib<br>Acalabrutinib<br>Olmutinib	DB09053<br>DB11703<br>DB13164	(Liang et al., 2018)
MEGF6	Oxaliplatin	DB00526	(Mou et al., 2021)
POSTN	Bevacizumab	DB00112	(Sun et al., 2025)
COL3A1	Cisplatin	DB00515	(Ren et al., 2024)

ligand–receptor (GPCR) pathways and from WNT pathway modulation add further *pan-cancer* support. GPCR activity and WNT drive plasticity, stem-like features, and immune escape in many tumors, and both have drugs in clinical use or trials (Domanska et al., 2013; Dorsam and Gutkind, 2007; Zhan et al., 2017; Harb et al., 2019). In parallel, enrichment of complement/coagulation and immune-lineage features outlines an inflamed, immune-rich tumor microenvironment often seen in *pan-cancer* studies, where coagulation and complement can support immunosuppression and spread of disease (Afshar-Kharghan et al., 2017; Falanga et al., 2013; Binnewies et al., 2018). Together, these results link RTK/GPCR input, calcium signaling, WNT programs, and the immune microenvironment into one *pan-cancer* picture. This agreement across multiple, druggable pathways shows that the computed gene set captures core and clinically relevant cancer biology (Monteith et al., 2017).

E.3.3. Analysis of drug dominance

To address the potential bias where the model might prioritize one drug over another (dominant-drug effect), we analyzed the stability of drug contribution scores across five independent experimental runs. We defined the Softmax Contribution as the average attention weight assigned to each drug branch before feature fusion.

As illustrated in Figure S5A, while the mean contribution across five experiments (5exp mean) suggests a bias toward Drug A, the individual experiments (exp_1 to exp_5) reveal significant heterogeneity. The dominance is not a fixed structural flaw but varies due to the stochastic nature of neural network initialization.

Furthermore, we investigated how this dominance affects the discovery of causal mechanisms. Using the combination of Ruxolitinib (Drug A) and Vismodegib (Drug B) as a case study, we analyzed the correlation between drug dominance and the enrichment of the Hedgehog signaling pathway (a known target of Vismodegib). We defined the dominance significance as the $-log10P - value$ of the difference between Drug A and Drug B contributions.

Figure S5B demonstrates a significant linear correlation ($k \approx -51.16$) between the dominance metric and the Normalized Enrichment Score (NES) of the Hedgehog pathway. This indicates that the model's ability to capture the specific mechanism of action (e.g., Vismodegib targeting Hedgehog) is dynamically linked to the attention weight that drug receives in a specific run. Consequently, instead of enforcing artificial symmetry, we suggest that users can leverage this property by selecting the specific experimental run where the drug of interest exhibits sufficient contribution, thereby enhancing the interpretability of that specific drug's mechanism.

References

Vahid Afshar-Kharghan et al. The role of the complement system in cancer. *The Journal of Clinical Investigation*, 127(3):780–789, 2017.

Eunus S Ali, Brototi Chakrabarty, Sarker Ramproshad, Banani Mondal, Neloy Kundu, Chandan Sarkar, Javad Sharifi-Rad, Daniela Calina, and William C Cho. TRPM2-mediated ca2+ signaling as a potential therapeutic target in cancer treatment: an updated review of its role in survival and proliferation of cancer cells. *Cell Communication and Signaling*, 21(1):145, 2023.

Johanna Andrae, Radiosa Gallini, and Christer Betsholtz. Role of platelet-derived growth factors in physiology and medicine. *Genes & Development*, 22(10):1276–1312, 2008.

Mikhail Binnewies, Edward W Roberts, Kelly Kersten, Vincent Chan, Douglas F Fearon, Miriam Merad, Lisa M Coussens, Dmitry I Gabrilovich, Suzanne Ostrand-Rosenberg, Catherine C Hedrick, et al. Understanding the tumor immune microenvironment (TIME) for effective therapy. *Nature Medicine*, 24(5):541–550, 2018.

Rand Gabriel M Buenaventura, Weiping Chen, Shuling Zhang, Beverly Mock, Glenn Merlino, and Yanlin Yu. Identification of PAEP as an immune activity modulator to mediate the immune resistance in tumor metastasis. *Cancer Research*, 84(6_Supplement):5524–5524, 2024.

Hui Chen, Zhiying Xu, Bin Yang, Xiaoli Zhou, and Hongfang Kong. RASGRF1 hypermethylation, a putative biomarker of colorectal cancer. *Annals of Clinical & Laboratory Science*, 48(1):3–10, 2018.

Liang Chen, Madison Dautle, Ruoying Gao, Shaoqiang Zhang, and Yong Chen. Inferring gene regulatory networks from time-series scRNA-seq data via GRANGER causal recurrent autoencoders. *Briefings in Bioinformatics*, 26(2):bbaf089, 2025.

Alissa J Cooper, Yoshihisa Kobayashi, Dewey Kim, Sarah E Clifford, Sasha Kravets, Suzanne E Dahlberg, Emily S Chambers, Jiaqi Li, Deepa Rangachari, Tom Nguyen, et al. Identification of a RAS-activating TMEM87A–RASGRF1 fusion in an exceptional responder to sunitinib with Non–Small cell lung cancer. *Clinical Cancer Research*, 26(15): 4072–4079, 2020.

Jean-Baptiste Demoulin and Ahmed Essaghir. PDGF receptor signaling networks in normal and cancer cells. *Cytokine & Growth Factor Reviews*, 25(3):273–283, 2014.

Urszula M Domanska, Roeliene C Kruizinga, Wouter B Nagengast, Hetty Timmer-Bosscha, Gerwin Huls, Elisabeth GE de Vries, and Annemiek ME Walenkamp. A review on CXCR4/CXCL12 axis in oncology: no place to hide. *European Journal of Cancer*, 49(1):219–230, 2013.

Robert T Dorsam and J Silvio Gutkind. G-protein-coupled receptors and cancer. *Nature Reviews Cancer*, 7(2):79–94, 2007.

Anna Falanga, Laura Russo, and Cristina Verzeroli. Mechanisms of thrombosis in cancer. *Thrombosis Research*, 131:S59–S62, 2013.

Anna Gaulton, Louisa J Bellis, A Patricia Bento, Jon Chambers, Mark Davies, Anne Hersey, Yvonne Light, Shaun McGlinchey, David Michalovich, Bissan Al-Lazikani, et al. ChEMBL: a large-scale bioactivity database for drug discovery. *Nucleic Acids Research*, 40(D1):D1100–D1107, 2012.

Mahmoud Ghandi, Franklin W Huang, Judit Jané-Valbuena, Gregory V Kryukov, Christopher C Lo, E Robert McDonald III, Jordi Barretina, Ellen T Gelfand, Craig M Bielski, Haoxin Li, et al. Next-generation characterization of the Cancer Cell Line Encyclopedia. *Nature*, 569(7757): 503–508, 2019.

Yayun Gu, Cheng Wang, Rongxuan Zhu, Jianshui Yang, Wenwen Yuan, Yanhui Zhu, Yan Zhou, Na Qin, Hongbing Shen, Hongxia Ma, et al. The cancer-testis gene, MEIOB, sensitizes triple-negative breast cancer to PARP1 inhibitors by inducing homologous recombination deficiency. *Cancer Biology & Medicine*, 18(1):74–87, 2021.

W Guo, R Liu, G Bhardwaj, JC Yang, C Changou, AH Ma, A Mazloom, S Chintapalli, K Xiao, W Xiao, et al. Targeting Btk/Etk of prostate cancer cells by a novel dual inhibitor. *Cell Death & Disease*, 5(9):e1409–e1409, 2014.

Yue Guo, Haitao Hu, Wenbo Chen, Hao Yin, Jian Wu, Chang-Yu Hsieh, Qiaojun He, and Ji Cao. SynergyX: a multi-modality mutual attention network for interpretable drug synergy prediction. *Briefings in Bioinformatics*, 25 (2):bbae015, 2024.

Xiaotian Han, Yechen Lu, Xiaoqi Li, Lingfang Xia, Hao Wen, Zheng Feng, Xingzhu Ju, Xiaojun Chen, and Xiaohua Wu. Overexpression of NPTX2 promotes malignant phenotype of epithelial ovarian carcinoma via IL6-JAK2/STAT3 signaling pathway under hypoxia. *Frontiers in Oncology*, 11:643986, 2021.

Jerry Harb, Pen-Jen Lin, and Jijun Hao. Recent development of wnt signaling pathway inhibitors for cancer therapeutics. *Current Oncology Reports*, 21(2):12, 2019.

Hanqing Hu, Meng Wang, Hongwei Wang, Zheng Liu, Xu Guan, Runkun Yang, Rui Huang, Qingchao Tang, Chaoxia Zou, Guiyu Wang, et al. MEGF6 promotes the epithelial-to-mesenchymal transition via the TGFβ/SMAD signaling pathway in colorectal cancer metastasis. *Cellular Physiology and Biochemistry*, 46(5):1895–1906, 2018.

Kexin Huang, Cao Xiao, Lucas Glass, and Jimeng Sun. Explainable substructure partition fingerprint for protein, drug, and more. In *NeurIPS Learning Meaningful Representation of Life Workshop*, Vancouver, Canada, 2019.

Lisa Hunihan, Dejian Zhao, Heather Lazowski, Man Li, Yuping Qian, Laura Abriola, Yulia V Surovtseva, Viswanathan Muthusamy, Lynn T Tanoue, Bonnie E Gould Rothberg,

et al. RASGRF1 fusions activate oncogenic RAS signaling and confer sensitivity to MEK inhibition. *Clinical Cancer Research*, 28(14):3091–3103, 2022.

Aleksandr Ianevski, Anil K Giri, and Tero Aittokallio. SynergyFinder 2.0: visual analytics of multi-drug combination synergies. *Nucleic Acids Research*, 48 (W1):W488–W493, 2020.

Delphine Ji, Zheng-wei Luo, Andrea Ovcjak, Rahmah Alanazi, Mei-Hua Bao, Zhong-Ping Feng, and Hong-Shuo Sun. Role of TRPM2 in brain tumours and potential as a drug target. *Acta Pharmacologica Sinica*, 43(4):759–770, 2022.

Binghan Jia, Xiaoling Zhao, Yao Wang, Jinlong Wang, Yingying Wang, and Yuemei Yang. Prognostic roles of MAGE family members in breast cancer based on KM-Plotter data. *Oncology Letters*, 18(4):3501–3516, 2019.

Tae Kyun Kim. T test as a parametric statistic. *Korean Journal of Anesthesiology*, 68(6):540–546, 2015.

Mengqing Li, Ling Wang, Yuting Zhan, Tingting Zeng, Xu Zhang, Xin-Yuan Guan, and Yan Li. Membrane metalloendopeptidase (MME) suppresses metastasis of esophageal squamous cell carcinoma (ESCC) by inhibiting FAK-RhoA signaling axis. *The American Journal of Pathology*, 189(7):1462–1472, 2019.

Rong Li, Jiao Gong, Cuicui Xiao, Shuguang Zhu, Zhongying Hu, Jinliang Liang, Xuejiao Li, Xijing Yan, Xijian Zhang, Danyang Li, et al. A comprehensive analysis of the MAGE family as prognostic and diagnostic markers for hepatocellular carcinoma. *Genomics*, 112(6):5101–5114, 2020.

Xiao Li, Sarah C Hughes, and Rachel Wevrick. Evaluation of melanoma antigen (MAGE) gene expression in human cancers using the cancer genome atlas. *Cancer Genetics*, 208(1-2):25–34, 2015.

Xiaoli Li, Nan Ding, Wenting Ma, and Ming Zhang. IFIH1-mediated post-transcriptional regulation of PTTG1 promotes proliferation and affects PHA-848125 sensitivity and prognosis in oropharyngeal carcinoma. *American Journal of Cancer Research*, 14(5):2157, 2024.

Chengyuan Liang, Danni Tian, Xiaodong Ren, Shunjun Ding, Minyi Jia, Minhang Xin, and Suresh Thareja. The development of bruton's tyrosine kinase (BTK) inhibitors from 2012 to 2017: a mini-review. *European Journal of Medicinal Chemistry*, 151:315–326, 2018.

Phillip Lippe, Sara Magliacane, Sindy Löwe, Yuki M Asano, Taco Cohen, and Stratis Gavves. Citris: Causal identifiability from temporal intervened sequences. In *International Conference on Machine Learning*, 13557–13603. PMLR, 2022.

Ruoqi Liu, Lai Wei, and Ping Zhang. A deep learning framework for drug repurposing via emulating clinical trials on real-world patient data. *Nature Machine Intelligence*, 3 (1):68–75, 2021.

Shuaibing Lu, Liangqun Peng, Fei Ma, Junhui Chai, Yawei Hua, Wei Yang, and Zhandong Zhang. Increased expression of POSTN predicts poor prognosis: a potential therapeutic target for gastric cancer. *Journal of Gastrointestinal Surgery*, 27(2):233–249, 2023.

Kang Luo, Yuanshao Zhu, Wei Chen, Kun Wang, Zhengyang Zhou, Sijie Ruan, and Yuxuan Liang. Towards robust trajectory representations: isolating environmental confounders with causal learning. In *Proceedings of the 33rd International Joint Conference on Artificial Intelligence*, 2243–2251, 2024.

Justin K Messex and Geou-Yarh Liou. Targeting BTK signaling in the microenvironment of solid tumors as a feasible cancer therapy option. *Cancers*, 13(9):2198, 2021.

Barbara A Miller. TRPM2 in cancer. *Cell Calcium*, 80:8–17, 2019.

Mirna Mina, Rebecca Conway, Reed Haga, Caleb Obregon, Purva Patel, Meredith Comstock, Erin Whiting, Jazmine Stubblefield, Edom Seyoum, Tarek Alhamami, et al. eP043: membrane metalloendopeptidase (MME) positively regulates phosphoinositide 3-Kinase (PI3K) signaling in triple negative breast cancer. *Genetics in Medicine*, 24(3):S29, 2022.

Gregory R Monteith, Natalia Prevarskaya, and Sarah J Roberts-Thomson. The calcium–cancer signalling nexus. *Nature Reviews Cancer*, 17(6):373–380, 2017.

Yueyang Mou, Nabin He, Mengyang Su, Zihua Zhong, Jiayu Ma, Jianling Liu, Xi'an Cheng, and Penggao Dai. MiR-1254 and MEGF6 regulates oxaliplatin resistance in human colorectal cancer cells. *American Journal of Translational Research*, 13(1):183, 2021.

Hyeon Jeong Oh, Jeong Mo Bae, Xian-Yu Wen, Nam-Yun Cho, Jung Ho Kim, and Gyeong Hoon Kang. Overexpression of POSTN in tumor stroma is a poor prognostic indicator of colorectal cancer. *Journal of Pathology and Translational Medicine*, 51(3):306–313, 2017.

Judea Pearl. Interpretation and identification of causal mediation. *Psychological Methods*, 19(4):459, 2014.

Judea Pearl, Madelyn Glymour, and Nicholas P. Jewell. Causal inference in statistics: A primer. 2016.

Judea Pearl et al. Models, reasoning and inference. *Cambridge, Uk: Cambridge University Press*, 19, 2000.

Kristina Preuer, Richard PI Lewis, Sepp Hochreiter, andreas Bender, Krishna C Bulusu, and Günter Klambauer. DeepSynergy: predicting anti-cancer drug synergy with deep learning. *Bioinformatics*, 34(9):1538–1546, 2018.

Friedrich Pukelsheim. The three sigma rule. *The American Statistician*, 48(2):88–91, 1994.

Na Qiang, Junjie Ao, Masato Nakamura, Keichi Katayama, Jiaqi Zhang, Tadayoshi Kogure, Keita Ogawa, Hiroaki Kanzaki, Ryuta Kojima, Keisuke Koroki, et al. MEGF6 knockdown ameliorates lenvatinib-induced muscle differentiation suppression and enhances the antitumor effect of lenvatinib on hepatocellular carcinoma. *Biochemical Pharmacology*, 235:116829, 2025.

Miran Rada, Nickolai Barlev, and Salvador Macip. BTK: a two-faced effector in cancer and tumour suppression. *Cell Death & Disease*, 9(11):1064, 2018.

Jiankun Ren, Songwei Zhao, and Junyu Lai. Role and mechanism of COL3A1 in regulating the growth, metastasis, and drug sensitivity in cisplatin-resistant non-small cell lung cancer cells. *Cancer Biology & Therapy*, 25(1):2328382, 2024.

Jin Roh, Su-Jin Shin, A-Neum Lee, Dok Hyun Yoon, Cheolwon Suh, Chan-Jeoung Park, Jooryung Huh, and Chan-Sik Park. RGS1 expression is associated with poor prognosis in multiple myeloma. *Journal of Clinical Pathology*, 70(3): 202–207, 2017.

Bernhard Schölkopf, Francesco Locatello, Stefan Bauer, Nan Rosemary Ke, Nal Kalchbrenner, Anirudh Goyal, and Yoshua Bengio. Toward causal representation learning. *Proceedings of The IEEE*, 109(5):612–634, 2021.

Nan Sethakorn and Nickolai O Dulin. RGS expression in cancer: oncomining the cancer microarray data. *Journal of Receptors and Signal Transduction*, 33(3):166–171, 2013.

Chao Shi, Xiaohan Wang, Jingping Li, Shang Wu, Zhihui Liu, Xiaofei Ren, Yunjiang Liu, et al. IFIH1 promotes apoptosis through the TBK1/IRF3 pathway in triple-negative breast cancer. *Neoplasma*, 71(6), 2024.

Yasuo Shiohama, Junya Ohtake, Takayuki Ohkuri, Daisuke Noguchi, Yuji Togashi, Hidemitsu Kitamura, and Takashi Nishimura. Identification of a meiosis-specific protein, MEIOB, as a novel cancer/testis antigen and its augmented expression in demethylated cancer cells. *Immunology Letters*, 158(1-2):175–182, 2014.

Damian Robert Sojka, Agnieszka Gogler-Pigłowska, Natalia Vydra, Alexander Jorge Cortez, Piotr Teodor Filipczak, Zdzisław Krawczyk, and Dorota Scieglinska. Functional redundancy of HSPA1, HSPA2 and other HSPA proteins in non-small cell lung carcinoma (NSCLC); an implication for NSCLC treatment. *Scientific Reports*, 9(1):14394, 2019.

Damian Robert Sojka, Sylwia Hasterok, Natalia Vydra, Agnieszka Toma-Jonik, Anna Wieczorek, Agnieszka Gogler-Pigłowska, and Dorota Scieglinska. Inhibition of the heat shock protein A (HSPA) family potentiates the anticancer effects of manumycin A. *Cells*, 10(6):1418, 2021.

Yongduo Sui, Xiang Wang, Jiancan Wu, Min Lin, Xiangnan He, and Tat-Seng Chua. Causal attention for interpretable and generalizable graph classification. In *Proceedings of the 28th ACM SIGKDD Conference on Knowledge Discovery and Data Mining*, 1696–1705, 2022.

Dongfeng Sun, Jie Lu, Hui Tian, Hao Li, Xiaozheng Chen, Feng Hua, Wenfeng Yang, Jinming Yu, and Dawei Chen. The impact of POSTN on tumor cell behavior and the tumor microenvironment in lung adenocarcinoma. *International Immunopharmacology*, 145:113713, 2025.

Ning Tang, Shasha Gai, and Qun Huang. A prognostic biomarker for bladder cancer correlated with immune infiltration is PAEP. *Journal of Biosciences and Medicines*, 12(4):185–209, 2024.

Ashish Vaswani, Noam Shazeer, Niki Parmar, Jakob Uszkoreit, Llion Jones, Aidan N Gomez, Łukasz Kaiser, and Illia Polosukhin. Attention is all you need. *Advances in Neural Information Processing Systems*, 30, 2017.

Bin Wang, Yue Pan, Yongjie Xie, Cong Wang, Yinli Yang, Haiyan Sun, Zhuchen Yan, Yameng Cui, Ling Li, Yaoyao Zhou, et al. Metabolic and immunological implications of MME+ CAF-mediated hypoxia signaling in pancreatic cancer progression: therapeutic insights and translational opportunities. *Biological Procedures Online*, 26(1):29, 2024.

Cheng Wang, Yayun Gu, Kai Zhang, Kaipeng Xie, Meng Zhu, Ningbin Dai, Yue Jiang, Xuejiang Guo, Mingxi Liu, Juncheng Dai, et al. Systematic identification of genes with a cancer-testis expression pattern in 19 cancer types. *Nature Communications*, 7(1):10499, 2016.

Jinxian Wang, Xuejun Liu, Siyuan Shen, Lei Deng, and Hui Liu. DeepDDS: deep graph neural network with attention mechanism to predict synergistic drug combinations. *Briefings in Bioinformatics*, 23(1):bbab390, 2022.

Wenjie Wang, Yang Zhang, Haoxuan Li, Peng Wu, Fuli Feng, and Xiangnan He. Causal recommendation: progresses and future directions. In *Proceedings of the 46th international ACM SIGIR Conference on Research and Development in Information Retrieval*, 3432–3435, 2023.

Yujian Wen, Jielong Huang, Shuhui Guo, Yehezqel Elyahu, Alon Monsonego, Hai Zhang, Yanqing Ding, and Hao Zhu. Applying causal discovery to single-cell analyses using CausalCell. *Elife*, 12:e81464, 2023.

Yutong Xia, Yuxuan Liang, Haomin Wen, Xu Liu, Kun Wang, Zhengyang Zhou, and Roger Zimmermann. Deciphering spatio-temporal graph forecasting: A causal lens and treatment. *Advances in Neural Information Processing Systems*, 36:37068–37088, 2023.

Chunjie Xu, Guangang Tian, Chunhui Jiang, Hanbing Xue, Manzila Kuerbanjiang, Longci Sun, Lei Gu, Hong Zhou, Ye Liu, Zhigang Zhang, et al. NPTX2 promotes colorectal cancer growth and liver metastasis by the activation of the canonical wnt/β-catenin pathway via FZD6. *Cell Death & Disease*, 10(3):217, 2019.

Fan Yang, Ling Lin, Xiaohua Li, Ronglan Wen, and Xin Zhang. Silencing of COL3A1 represses proliferation, migration, invasion, and immune escape of triple negative breast cancer cells via down-regulating PD-L1 expression. *Cell Biology International*, 46(11):1959–1969, 2022.

Jie Yang, Guoliang Wu, Qian Wu, Xinming Hu, Zechun Peng, Dan Zhang, and Shuming He. TMB-related immune gene PAEP influences clear cell renal cell carcinoma progression and prognosis. *European Journal of Medical Research*, 31 (1):280, 2026.

Lin Yu, Ying Luo, Di Wu, and Shiyuan Hong. Effect of RNA sensor IFIH1 on proliferation, migration and invasion of HPV positive cervical cancer cells. *Journal of Army Medical University*, 44(16):1651–1660, 2022.

Yue Yu, Chang-Ming Tan, and Yuan-Yuan Jia. Research status and the prospect of POSTN in various tumors. *Neoplasma*, 68(4), 2021.

Bulat Zagidullin, Jehad Aldahdooh, Shuyu Zheng, Wenyu Wang, Yinyin Wang, Joseph Saad, Alina Malyutina, Mohieddin Jafari, Ziaurrehman Tanoli, Alberto Pessia, et al. DrugComb: an integrative cancer drug combination data portal. *Nucleic Acids Research*, 47(W1):W43–W51, 2019.

X Zeng, SC Sikka, L Huang, C Sun, C Xu, D Jia, AB Abdel-Mageed, JE Pottle, JT Taylor, and M Li. Novel role for the transient receptor potential channel TRPM2 in prostate cancer cell proliferation. *Prostate Cancer and Prostatic Diseases*, 13(2):195–201, 2010.

Lu-lu Zhai, Pei-pei Qiao, Yue-Shen Sun, Zhi-Gang Tang, and Tong-Fa Ju. Upregulated HSPA2 predicts early relapse of pancreatic cancer after surgery. *Gland Surgery*, 10(7):2140–2149, 2021.

Tailan Zhan, Niklas Rindtorff, and Michael Boutros. Wnt signaling in cancer. *Oncogene*, 36(11):1461–1473, 2017.

Hanyu Zhang, Cheng Ding, Yatong Li, Cheng Xing, Shunda Wang, Zhangping Yu, Lixin Chen, Pengyu Li, and Menghua Dai. Data mining-based study of collagen type III alpha 1 (COL3A1) prognostic value and immune exploration in pan-cancer. *Bioengineered*, 12(1):3634–3646, 2021.

Huaiwu Zhang and Jing Tang. CADS: causal inference for dissecting essential genes to predict drug synergy. In J. Tang, X. Lai, Z. Cai, W. Peng, and Y. Wei, editors, *Bioinformatics Research and Applications*, volume 15756 of *Lecture Notes in Computer Science*, 288–300. Springer, Singapore, 2025. doi: 10.1007/978-981-95-0698-9_24.

Ling Zhang, Jun Gao, Zhaoshen Li, and Yanfang Gong. Neuronal pentraxin II (NPTX2) is frequently down-regulated by promoter hypermethylation in pancreatic cancers. *Digestive Diseases and Sciences*, 57(10):2608–2614, 2012.

Shao-wei Zhang, Nan Zhang, and Na Wang. Role of COL3A1 and POSTN on pathologic stages of esophageal cancer. *Technology in Cancer Research & Treatment*, 19: 1533033820977489, 2020.

Siyang Zhang, Han Wang, Jiao Liu, Tao Tao, Zhi Zeng, and Min Wang. RGS1 and related genes as potential targets for immunotherapy in cervical cancer: computational biology and experimental validation. *Journal of Translational Medicine*, 20(1):334, 2022.

Zhaohui Zhu, Hanlu Wang, Xu Qian, Meiling Xue, Aijun Sun, Yifei Yin, Jinhai Tang, and Jian Zhang. Inhibitory impact of cinobufagin in triple-negative breast cancer metastasis: involvements of macrophage reprogramming through upregulated MME and inactivated FAK/STAT3 signaling. *Clinical Breast Cancer*, 24(4):e244–e257, 2024.
